# Dynamic Bayesian Networks, Elicitation, and Data Embedding for Secure Environments

**DOI:** 10.3390/e26110985

**Published:** 2024-11-17

**Authors:** Kieran Drury, Jim Q. Smith

**Affiliations:** Department of Statistics, University of Warwick, Coventry CV4 7AL, UK; j.q.smith@warwick.ac.uk

**Keywords:** Bayesian networks, dynamic Bayesian networks, decision support systems, expert judgement, elicitation, model libraries, missing data, crime intervention, causality

## Abstract

Serious crime modelling typically needs to be undertaken securely behind a firewall where police knowledge and capabilities remain undisclosed. Data informing an ongoing incident are often sparse; a large proportion of relevant data only come to light after the incident culminates or after police intervene—by which point it is too late to make use of the data to aid real-time decision-making for the incident in question. Much of the data that *are* available to the police to support real-time decision-making are highly confidential and cannot be shared with academics, and are therefore missing to them. In this paper, we describe the development of a formal protocol where a graphical model is used as a framework for securely translating a base model designed by an academic team to a fully embellished model for use by a police team. We then show, for the first time, how *libraries* of these models can be built and used for real-time decision support to circumvent the challenges of data missingness seen in such a secure environment through the ability to match ongoing plots to existing models within the library.The parallel development described by this protocol ensures that any sensitive information collected by police and missing to academics remains secured behind a firewall. The protocol nevertheless guides police so that they are able to combine the typically incomplete data streams that are open source with their more sensitive information in a formal and justifiable way. We illustrate the application of this protocol by describing how a new entry—a suspected vehicle attack—can be embedded into such a police library of criminal plots.

## 1. Introduction

Particularly since the turn of the millennium, the application of probabilistic models, especially graphical models (see, e.g., [1,2,3]) to the prevention of criminal activities has been of very high interest. Such models have been developed to provide police with probabilistic predictive abilities for use within various orchestrated criminal activities. For example, Ref. [4] details how influence diagrams stemming from engineering risk analysis methods can be constructed to support decision-making over a choice of possible terrorism countermeasures; Ref. [5] develops Bayesian network (BN) models representing the cognitive decision-making process of police officers in evaluating possible “terrorism-centric behaviours”; and Ref. [6] proposes a data-driven approach to learn the causal structure of a Bayesian network which describes the real-world system surrounding maritime terrorism activities. However, in this paper, we focus on the development and use of the type of Bayesian hierarchical model seen in [7] that describes how a generic suspected terrorist plot is expected to develop. We describe the adversarial causal algebra behind the use of this causal model in frustrating the progress of a suspected plot and explain how police can use such a model to provide Bayesian decision support to determine the predicted efficacy of potential real-world interventions on the suspected plot. The main development in this paper is two-fold. First, we explain how an academic team and an in-house police team can work together on the co-creation of these Bayesian hierarchical models, expressed here as a two time-slice dynamic Bayesian network (2TDBN), while ensuring any secure information is kept completely unknown to the academic team. The second main contribution in this paper is the description of a protocol for creating a *library* of these models—a securely-kept bank of models of terrorist plots from past cases that can then be used to aid the rapid development of models for newly discovered unfolding plots.

Typically, the domains of terrorist activities we consider are very fast moving and various components of critical informative data streams are often systematically missing. Therefore, especially as we are concerned with the construction of models for specific, individual, ongoing plots, we cannot simply adopt such a data-driven approach as seen in [6]. The missingness of data seen here is usually in no sense at random and often comes as a result of data relevant to an ongoing criminal plot becoming available only after the plot has either been carried out or prevented. Data that are useful for guiding police in the intervention of such plots often cannot be observed by police at the point where they must decide whether to intervene, usually appearing after this point in time in the form of court hearings and incident reports. These issues often require models to be customised in subtle ways so that predictive algorithms are fit for purpose [7]. Further, as the combination of these two issues leads to a limited ability to utilise data within the model construction, there is an increased reliance on the use of elicitation methods (see, e.g., [3,8,9]). When these elicitation methods are performed in a properly structured manner to avoid many of the cognitive biases that can significantly hinder the accuracy of experts’ judgements [10], this process can add significant delays to the model creation timeline. In such a fast-moving domain, this impact can be catastrophic as police have limited time before they must intervene in an ongoing plot. For several reasons therefore, police strongly benefit from being able to match a current ongoing plot to a pre-existing embellished probability model from a previous incident which can be then adjusted to suit the ongoing plot as fit. This process is aided through the use of *libraries* which we discuss in this paper as a mechanism to tackle the type of missingness of data that we see in this application.

Yet there exists an even bigger challenge. When performing this modelling task, police must ensure that the types and extents of the various streams of data available to them, and the precise algorithms they use to make inferences are not directly betrayed to a suspected criminal who could then use this information for their own advantage. So any operationalisable decision analysis based on these probabilistic graphical models typically needs to be undertaken *securely* behind a firewall where police knowledge and capabilities are kept secret. Therefore, data that would otherwise inform police behind the firewall of the critical features of the underlying process are not only systematically missing or disguised, but must be kept secret from—and are therefore missing to—any remote team of specialist decision analysts and statistical modellers supporting police in developing decision models. These academic specialist modellers are typically required for the development of advanced, bespoke models of the kind police will need for effective decision support. Typically, police only have limited in-house modelling resources. It is therefore of the highest importance to develop a co-creation protocol that enables the sharing of both academic expertise and police intelligence securely across the firewall to avoid the many problems associated with the lack of experience police teams may have in developing these probabilistic models, as well as with the sources of data missingness encountered by both teams in the ways described above.

Such protocols allow police to directly apply state-of-the-art inferential and elicitation methodologies to systematically address and surmount these very particular challenges that they would otherwise struggle to overcome. The protocols we report here—to develop co-created libraries of customised graphical models—have now been successfully applied by co-creation teams across a wide range of secure use cases. The co-creating teams begin by building generic frameworks that *describe and categorise* incidents of crime. Policing agents are then trained by academic teams to match an ongoing incident to a particular graphical model. The structural information embedded in each graph enables the academic team to guide police in building a library of embellished probability models around these frameworks which can then support police in frustrating crimes that are planned to harm the general public. In this paper, we focus on those police libraries where the appropriate choice of structural framework, including that seen in [7], for each category of unfolding criminal incident, can be represented by the graph of a Bayesian Network. Each graph is then developed into a full probability model through specification of the quantitative relationships between its nodes (see, e.g., [3]). When assuming the BN to be discrete, as we do in this paper, this involves populating the conditional probability tables (CPTs) for each node in the network. The protocol we have been developing over the last ten years is reported here for the first time and provides a generic yet detailed methodology for this.

A central concept behind this protocol is the synergetic communication across the *parallel inferential code* on either side of a secure firewall. Thus, using open-source data, an academic team of analysts outside the firewall first elicit families of probability models consistent with generic developments associated with particular categories of crime. Through a co-creation scheme of sequential interactions with a parallel team of police, the academic team then incrementally builds up a library of coded-up probability models—one for each category of crime—each described by its own BN. Parallel coded models simultaneously developed by the two teams are enhanced by the shared structure of BNs on either side of the firewall, as it is often the *quantitative embellishment* of the BN that is so sensitive to the secure intelligence inaccessible to the academic team. These then provide the framework for symbiotic embellishments in ways we describe below. Using the Bayesian paradigm, these embellishments then enable police to predict the progress of new incidents as these unfold—both when simply observing the incident and when considering intervening in various ways. These predictions can then be used to inform a decision support system designed to frustrate the progress of the crime and mitigate its potential harm.

The co-creation protocol we describe here enables defenders to efficiently and effectively communicate with professional decision support experts—who are not necessarily sufficiently security cleared—to nevertheless guide the development of an appropriate probabilistic model. In particular, police, guided by an academic team, are able to maintain the security of sensitive information to build up their own secure libraries of BNs.

Within the context of policing, it is clearly essential that the graphical framework provides not only predictions of what might happen if the police simply watch the progress of a crime, but also what might happen if they intervene. In technical terminology, this requires the BN to be causal. In Section 2, we introduce our running example of the modelling of a suspected vehicle attack before giving a brief review of such causal challenges as these might apply to this particular type of adversarial domain. We also describe how a Bayesian decision analytic paradigm provides a natural framework for such a secured co-creation. Then, in Section 3, we describe the types of libraries of graphical probability models that are currently being developed, focusing on describing the development of a library of a particular class of graphical models called plot models. We also describe how a library may be developed for our running example of vehicle attacks.

In Section 4, we proceed to detail a protocol we have developed through our experiences developing this kind of library. This preserves the security of the data, missing to the external guiding team of academics, whilst still providing a conduit for fast technological transfer from the academic team to the police. In Section 5, we discuss, in more technical detail, why this application of graphical Bayesian modelling is feasible and the circumstances under which formal inferences across the whole library can be applied to enhance the propriety of this technological transfer. In particular, we outline how partial information retrieved from collections of past incidents can sometimes be covertly applied by police to a current incident in an entirely formal and justifiable way. The BN framework thus enables academics to guide police in their accommodation of the types of systematic missingness of informedly censored data that often dominate the open-source information space. This also presents a systematic methodology for building and relying upon libraries of models for different incidents of recurring types of plots to overcome the challenges faced as a result of data about ongoing plots not being readily available to support real-time decision-making. We end the paper with a discussion of future research and encountered challenges.

## 2. Bayesian Analyses for Secure Domains

### 2.1. A Running Example—A Suspected Vehicle Attack

We begin by describing a scenario that acts as the foundation to our running example throughout the paper. This scenario aims to demonstrate why a police team would need to undertake such a Bayesian decision analysis as we allude to in this paper, and further aims to explore how a police team would utilise the plot models that we describe here in order to evaluate each of their options for intervening in the progress of a suspected plot.

Our running example concerns a police team who have reason to believe a known suspected criminal is plotting a vehicle attack in central London with the aim to provide significant harm to members of the general public. We first note that the model we refer to for this vehicle attack plot has been reported in the open literature (see, e.g., [7]) and can thus be shared in this work. The police team know from previous cases, and through known intelligence about how such a plot must be planned and later carried out, that there are a series of *phases* through which the plot must pass before the attack itself can occur. In this paper, we provide the following generic sequence of phases which fits several types of attack, including vehicle attacks, without compromising any secure intelligence police have from real cases. Other types of attacks that this sequence of phases fits include firearms attacks, knife attacks, crossbow attacks, and bomb attacks. The generic sequence of phases can be listed as follows: $w_{1}$—recruited to the plot; $w_{2}$—training to be capable of perpetrating the plot; $w_{3}$—identifying an appropriate target of the plot and reconnoitring it; $w_{4}$—obtaining the equipment needed to attack; and $w_{5}$—travelling to the target to make the attack. We further introduce the phase $w_{0}$—not engaged, representing the abortion of the plot by the suspect. We may choose to define a finer or a more specific sequence of phases for the modelling of the vehicle attack, though it can be beneficial to define attributes in a model as generally as possible (without sacrificing model quality) such that the model can be easily translated and modified for other similar cases (see, e.g., [11]). Further assumptions about the nature of such a plot are described in Section 3.4.1 and are translated into structural features of the graphical model.

It is clear that the plotted vehicle attack is likely to be successfully perpetrated if the police team do not intervene to frustrate the progress of the plot. Intervention is not guaranteed to foil the plot, though it should reduce the likelihood of perpetration and thus reduce the likelihood of harm to the general public. Therefore, it is clear that the police team will deem it necessary to intervene. However, they will have several interventions available to them. Each potential phase of the plot will carry with it different intervention options for the police—if not in the actual method of real-world intervention, then in the likelihood of the intervention successfully preventing the vehicle attack from being carried out. The police team thus need to evaluate both when and how to intervene in the progress of the plot. There are further difficulties in that police often do not know with certainty which phase the plot is currently in, and in not always having sufficient data to model this with a high degree of accuracy. In order to both model the progress of the plot and to be able to model the likely outcomes of any police intervention, it is necessary to develop a causal Bayesian hierarchical model of the type we describe throughout the remainder of this section as well as in Section 3.

### 2.2. A Probabilistic Foundation for Framing Protocols

Historically, a common way for policing bodies to commission help in building probability models of sensitive domains has been to simply guess what an incident in a more benign domain analogous to a sensitive modelling problem of interest might be. Then, by copying the methodology which applies to this benign domain, the police build their own in-house model for their more sensitive applications. Of course, matching across domains in this way is perilous for any party who only partially understands the academic domain they match to, and such a match may not exist. Even if a good match to the secure domain is successfully identified, the subsequent transfer of technology is often in practice naïve because that transfer has needed to be exploited entirely in-house and is open to little quality control. This has limited the success of the technology transfer of frontier inferential methodology onto critical, sensitive policing domains.

However, more recently within the UK and elsewhere, a more direct transfer of cutting-edge statistical and machine learning technologies, modelling sensitive applications, has been commissioned. This has involved a sustained nurturing of suitable academic relationships across a firewall. A technical team within the relevant policing organisation work symbiotically and in real time alongside an academic team. The academics learn as much as they can about the domain through open-source information and create a model from this. They then translate this particular model and associated inferential methodologies—*bespoke to this particular domain*—to help police build a customised probability model suitable to inform the decision support tools they need to protect the public from harm.

The fact that the academic team understands the actual domain of interest as far as security allows means that they can not only help police template their models so that they are fit for purpose, but also—through understanding the types of algorithms that police are using—that they can provide vital ongoing support to ensure an appropriate calibration and adaptation of these models as the crime environment develops. We note that a significant recent investment of resources within UK policing organisations has now ensured there are sufficient in-house skills available to make such co-creation possible.

Although such co-creation projects for fast technological transfer continue to improve, through active engagement over a number of years and over a diverse set of domains, we have been able to begin to develop protocols through which this symbiosis might work. It is therefore timely to share these. This paper describes one such development—the building of graph-based decision support systems to pursue criminal plots.

The particular approach we describe here is a subjective Bayesian one. There are a number of reasons for this choice:

1. Bayesian decision support systems are *prescriptive* in nature. These therefore almost automatically carry with them a systematic framework around which a protocol for technological transfer we have outlined above can be performed.
2. Bayesian methodologies are now *widely developed* and arguably provide the best modelling framework for a spectrum of different challenging inferential settings. More specifically, these have now been successfully applied across a myriad of complex domains very similar to various secure domains on to which technologies need to be transferred.
3. Bayesian methods *interface well with cutting-edge data analytic methods*—currently being developed in both computational statistics and machine learning communities.
4. A critical feature of many secure environments is that the streaming time series and historic data often suffer from being systematically missing, disguised and, for some central variables, completely latent. This is typically the case for high-threat criminal plots for which data about the plot are usually only observed and become available after the plot is carried out or prevented. This usually demands that expert judgements (see, e.g., [3,9]) need to be explicitly embedded into models before these are fit for purpose. The Bayesian paradigm provides *a formally justifiable way for embedding such necessary expert judgements* into that inferential framework through the use of priors that can be updated with further judgements and data as they become available [2].
5. Once such prior probabilities are elicited, Bayes Rule and graphical propagation algorithms [2] can be embedded in the code to update police beliefs about the underlying processes, even when that data are seriously contaminated or disguised, in ways we illustrate in this paper. In this way, Bayesian methodology transparently informs and helps police *adjust their current beliefs* in terms of what they observe which, as well as being consistent with the way they make inferences, *puts the police centre stage*. The approach therefore helps them *own* the support given by the academic team.

Recall that to perform a Bayesian decision analysis (see, e.g., [2]), for each *decision* d∈D—where henceforth the decision space D includes the option $d_{\phi }$ of doing nothing—the policing organisation calculates their subjective expected utility (SEU) $\bar{U}\left(d\right)$ to identify the option $d^{*}\in D$ that maximises this function. When the police team have a utility function U(a) on a vector of attributes a, and $p_{d}\left(a\right)$ denotes their probability density over their attributes a, these SEU scores are given by

(1) $$\bar{U}\left(d\right)≜\int U\left(a\right)p_{d}\left(a\right)da$$

In a criminal setting, the attributes a will typically include measures of harm to the public as well as measures of the policing resources required to counter this threat in various ways. Within this paper, we will assume, for brevity, that these attributes have been elicited and are known to all parties. Discussions of how each attribute could be elicited have now appeared elsewhere [12,13]. Under this assumption, police will then simply need guidance to appropriately construct the joint probability mass functions $\left\{p_{d}\left(a\right):d\in D\right\}$—the subject of the remainder of this paper—so that they can calculate the scores $\left\{\bar{U}\left(d\right):d\in D\right\}$ (from Equation (1)) needed to score the efficacy of any potential interventions they might make to mitigate harm to the public.

Usually, police will need to learn about a indirectly through gathering information about a much longer random vector $\xi =\left(\xi_{1},\xi_{2},\ldots ,\xi_{n}\right)$ with probability density mass functions $\left\{p_{d}\left(\xi \right)\in P_{G}:d\in D\right\}$. Such a vector for our vehicle attack example may include features such as the number of visits to radical websites, the number of visits to potential target locations, and the number of visits to online vehicle dealership pages [7]. The components of ξ measure the critical explanatory features of the underlying process of the criminal activity as understood by police. Although our protocols apply more widely, for simplicity—and for consistency with the topic of this special issue—we will assume in the first instance that the relationships between the nodes of the vector ξ can be described by a two time-slice dynamic Bayesian network (2TDBN) [3]. Note that it is trivial to check that any 2TDBN with a finite number of time steps can be written as a probabilistically equivalent BN by displaying separate nodes for each variable at each time step, and drawing edges for time-lag relationships, as well as for those existing in the static structure.

### 2.3. Eliciting a Bayesian Network for Sensitive Domains

#### 2.3.1. Four Elicitation Steps and Some Causal Hypotheses

When the vector ξ is not short, applied Bayesian modellers have discovered that it is wise to build $\left\{p_{d}\left(\xi \right):d\in D\right\}$ in four steps. The underlying intuition behind the practice of these steps can be seen in [14]. Here, we build on this work by providing these four steps explicitly. The first step is to elicit those variables that best describe the criminal process in focus. This step is often missing in standard BN analyses of data-rich environments where the interest lies solely in making inferences about the relationships between various *prespecified* measurements. However, when describing a type of crime, this step is critical; much of the expert judgements from police about how crimes unfold is embedded in the choice of the components ξ and forms a central theme within the co-creating protocol we describe below. These critical features ξ—usually latent to the police team—will be used to frame hypotheses about what might actually be happening within an unfolding current suspected incident. But police will often only be able to observe the out-turns of these features, only knowing a task is being performed once it is complete. Available data do not usually directly inform these features, and, when they do, they are often ephemeral and disguised. Furthermore, even what can be observed, often by its very nature, cannot be shared with the academic co-creation team without betraying secret police capabilities. On the other hand, interpretable and explainable latent variables—becoming some of the components of ξ—can usually be freely shared because these and their relationships are generic, and data and expert judgements informing these are typically available in the open-source social science domain.

Secondly—once the components of ξ have been identified—the academic team can begin to elicit structural prior information from police about the likely types of relationships between them, here assumed expressible as a BN G whose vertices are the components of ξ. Again, because of the paucity of data on many complete past incidents across the whole system, a class of possible BNs are usually elicited *before* accessing past datasets. Then, using standard Bayesian technologies, any informative data streams can be subsequently used to adjust such prior structural hypotheses. This structural elicitation phase then forms the first stage of the domain elicitation. The graphical model structure ensures that domain expertise, predominantly expressed through natural language by police, can be embedded at the very core of the stochastic model by the academic team. This critical faithful qualitative information is supported by reasoned arguments, and so is typically much less ephemeral than its probabilistic embellishments and is securely placed at the heart of the police team’s predictive models. This is especially important both in classifying different categories of incident and for describing the types of driving processes behind individual incidents within each category.

The third stage of the elicitation process is then for the academic team to guide police in embellishing this qualitative model into a set of full probability models needed for a Bayesian decision analysis. This is conducted by first eliciting prior information—here about the probability that a particular BN best describes a particular class of plot and then the particular CPTs of each given category of incident, given each type of suspect and environment, and each possible intervention police might make. These expert judgements are then calibrated against any available historical data relevant to the category of crime being set up—often sadly sparse for many of its CPTs. For example, CPTs capturing the intent and capability of a suspect are usually a central part of a BN of an unfolding crime. However, a criminal’s intent will only be fully known by the perpetrator and data about the capabilities of any particular suspect are likely to be missing. This is why probabilistic expert judgements from criminologists and police, and appropriate statistical models, need to inform such tables. Of course there will be considerable data collected on each past case that inform some of the entries in the CPTs of the relevant BNs. So, once these expert judgements are in place, such probability judgements can be further refined using the usual Bayesian machinery designed to do this.

Thus, only once this probability model is in place are the final conditional probabilities elicited that are needed to complete the CPTs for the current incident involving a particular triaged suspect—the fourth step in the process. Then, using this probability model as a prior for the unfolding incident, any available streaming data collected that inform the current incident can be used to update the predictives about the progress and potential outcome of the incident currently being policed. This is achieved simply through police using customised propagation algorithms matched to those provided by software transferred by the academic teams using their parallel, less-informed BN and its CPTs.

#### 2.3.2. Causality and Libraries of Crime Models

One critical issue is, as far as is possible, for the academic team to try to ensure that the elicited BNs of each category of criminal process are *causal*. Fortunately, BN representations of causal processes have been widely studied for a number of decades, both from a foundational and methodological perspective beginning with seminal work by [1,15]. The majority of this development has focused on the construction of causal discovery algorithms (see, e.g., [16,17]). However, the established reasoning machinery it utilises can also be applied to build causal Bayesian models of the type we need here [2,3]. In particular, these causal models we develop are rooted in an adjusted version of Pearl’s structural causal model (SCM) framework [1]. We adjust the SCM framework by retaining Pearl’s definition of an SCM and applying the same principles forming his do-calculus approach to interventions on the model, but further accounting for the adversarial nature of the crime-based system being intervened on. This is referred to as *intelligent causation* and allows us to consider how the external world—as seen by police to include suspected criminals—may change after an intervention by police, including changes caused by suspects themselves [13].

To apply causal reasoning to criminal processes in the way we introduce above, we argue in [13] that it is helpful to embed further properties of a model which might justify it being described as truly causal according to this adversarial SCM framework. These properties were demanded in general long ago by [18] but have largely been ignored until recently by the graphical machine learning community. The three causal properties—additional to Pearl’s SCM construction—that we demand in the construction of a BN that models a particular category of crime we discuss in this paper are given below:

1. The BN provides a framework not only for police predictions about what will happen if they do not intervene, but also if they do [1,2,15]—including how targeted suspected criminals are expected to react upon discovering such an intervention against them is being implemented by police [13]. This is an adversarial form of *interventional causality*.
2. The chosen BN provides a template for the way many different crimes within a given category might unfold. So this must have this type of generic quality called *causal consistency* in [18]. We note that this type of concept has recently reappeared in a rather different form as abstraction transport [19].
3. To double guess a criminal’s reactions to an intervention they might make, it would be helpful if the police team tried to ensure that the structural beliefs expressed through the graph were shared by the criminal [13]. This is a strengthened version of the long-established *coherence* property that we refer to as *causal common knowledge*.

In a causal model as described by the first bullet above, the structural framework of its BN will be shared for all $\left\{p_{d}\left(\xi \right):d\in D\right\}$ and, by the second bullet, for any given incident within the category. We note that the first invariance property is now widely hypothesised for BNs (including 2TDBNs) in order to make inferences about, for example, the efficacy of treatment regimes, albeit in a non-adversarial setting (see, e.g., [3]).

Such causal assumptions are required of the models we demonstrate in this paper in order for the use of these models by police to be valid. Specifically, in this domain of crime intervention, the police team wish to evaluate the consequences of potential attempts to prevent a plot that is suspected to be in some active phase. Therefore, the main use of these models is in applying interventions *predictively* and analysing the model outputs to determine the predicted efficacy of such interventions. By intervening in the model in this way, we are implicitly assuming that our network is causal in the technical sense described above. It is argued in [1] that the network being causal is what allows it to act as a vehicle for representing and reacting to external changes—in this case, those changes are the police interventions affecting the progress of a plot. If the network is not causal and does not satisfy the assumptions above, it is not logical to be able to evaluate the consequences of such external interventions in the model of a suspected plot. Therefore, if we construct and apply standard BNs, the model outputs cannot be reliable in providing decision support.

It is worth noting that we focus on these police interventions from this predictive perspective rather than from a counterfactual perspective. The former concerns the consequences of intervening in the system in the present and immediate future, while the latter concerns questions about what would have happened in the system had a particular attribute taken a different value. Clearly, police teams wish to evaluate their potential interventions predictively as they are utilising such a model to intervene in an unfolding plot. In using counterfactuals to evaluate the efficacy of possible interventions, they would be implicitly assuming that one model will apply to multiple individual cases. Even if this were the case, this model could then simply be used to evaluate interventions predictively for each case. Therefore, we focus on the evaluation of interventions for this domain through a predictive lens. There has been a long course of conversation and debate about causal inference without the use of counterfactuals in this way, notably in [20] and subsequent discussions.

The most studied class of interventions $d_{I(\hat{\xi })}\in D$ associated with an interventionally causal BN are ones that force the measurement $\xi_{i}$ to take the value ${\hat{\xi }}_{i}$ for each $i\in I\subseteq \left\{1,2,\ldots ,n\right\}$ with $\hat{\xi }≜\left\{{\hat{\xi }}_{i}:i\in I\right\}$—interventions commonly referred to as “doing” $\left\{\xi_{i}={\hat{\xi }}_{i}:i\in I\right\}$ (see, e.g., [1,2,15]). Then the valid BN for the intervention is one which simply substitutes the CPT of the conditional mass function of $\xi_{i}$ with one that assigns probability one to the event $\left\{\xi_{i}={\hat{\xi }}_{i}\right\}$ irrespective of the parent configuration whilst leaving all other CPTs the same as they were when there was no intervention. Pearl calls a BN G *causal* when these BNs, perhaps embellished with different collections of CPTs within the same directed acyclic graph (DAG) G, $d_{I(\hat{\xi })}\in D$, are all valid assertions about a given domain [1]. Then, the joint density $p_{dG}\left(\xi \right)$ for all $d_{I(\hat{\xi })}\in D$ can be factorised as

(2) $$p_{dG}\left(\xi \right)=\prod_{i=1}^{n}p_{id}\left(\xi_{i}|\xi_{Q(i)}\right)$$

where $\xi_{Q(i)}$ is the set of parents of $\xi_{i}$, i=1,2,…,n in G. Within the adversarial setting of criminality, such vanilla assumptions will not usually hold for a simple BN of the unintervened process [13]. However, we demonstrate in [13] that—provided the BN is chosen to be rich enough to model the capability of the criminal and their intent, and what the criminal might be able to learn about how the intervention might be made—then the same DAG structure and analogous substitution rules can be used. Police will then simply substitute some of the CPTs valid when no intervention is made for others when implementing $d\neq d_{\phi }$, d∈D. For example, police may intervene by restricting access to, or preventing completion of, a heavy goods vehicle driving course. This would be modelled by replacing any conditional probability of completion of this task with a zero, assigning probability one to the task remaining incomplete despite what values any parent nodes may take. The particular substituted CPTs determined by *d* can be assumed valid in this adversarial context. Examples of the precise nature of such substitutions is beyond the scope of this paper but can be found in [13].

This interventional causality property for a BN model of a policing application can be particularly useful because the same BN can then be used as a predictive framework whether or not they choose to intervene to try to prevent a crime from being committed. We note that the BN elicitation protocols developed by [3] try to ensure that this type of causal property is automatically embedded within the model. For the purposes of this paper, we henceforth assume that this type of causal property is co-created by the two teams for all interventions d∈D that police might consider making for a criminal process described by its graph G.

For the purposes of building a *library of BNs* for particular categories of crime that can then be used to match similar yet distinct incidents, we also need to demand both the *consistency* and *coherence* properties that were first demanded of causal systems by [18] and are here applied to BNs. So we require that the elicited BN will *remain a valid template for many analogous instances* of crime within the same category [13,14]. A necessary skill of academic teams is the ability to ensure that categories are defined sufficiently finely so that they are similar in this structural sense such that the protocol below applies.

The final property we also may need to use when applying these models to predict the consequences of a police intervention is one based on the game-theoretic notion of common knowledge. Bradford Hill [18] demanded that the hypotheses embodied by a causal model should, on reflection, appear at least plausible to other intelligent people. In [13], we strengthen this hypothesis and then apply it to a suspect assumed by police to be intelligent. So, in particular, inferences police make hypothesise that the suspected criminal is *intelligent and shares the same understanding as the police team about how their planned crime might be successfully perpetrated*. We note that this hypothesis is only needed if the suspect can learn that police have committed to intervene in a particular way and can react to this intervention. This is indeed often a true characteristic of criminal activities in practice. Examples of such visible interventions are police raids or the establishment of protections for potential targets. The plausibility of this assumption needs to be tested on a case-by-case basis; in our running example, it is almost automatic. We demonstrate in [13] through a number of examples how this hypothesis facilitates police, guiding them in double guessing how a criminal might react after hearing that a particular intervention has been put in place, and therefore guiding them in producing the necessary forecast distributions associated with applying interventions that become visible to a criminal.

Most sensitive policing domains are ones in which a crime develops dynamically. However, the BN framework extends into a model of evolving domains where, in particular, analogous simple “do” algebras are especially simple to define for the dynamic Bayesian network (DBN) G over time steps t=0,1,…,T that we use in our running example. This is because any DBN G is equivalent to a BN with graph $G^{+}$ whose vertices are time indexed. Its irrelevance statements are therefore implicit in the DBN—see e.g., [3] for a precise definition of this construction. We can now duplicate the algebra defined above on $G^{+}$ and translate this to G. So such maps fall within our generic framework—albeit with often a massive set of factors and types of interventions—for example when an intervention might be applied and for how long, as illustrated in [13].

### 2.4. Data and Information in Secure Domains

Under recent co-creation schemes with policing agencies, it has been possible for academics to help in-house domain experts to better design probabilistic models of serious crime. However, of course, these academics still only have restricted information about the domains of application. Several of the model’s attributes will have no data available, either from historic analogues or within the currently monitored case, to directly inform their CPTs, and a lot of the data that *are* available to the police must necessarily be kept secure behind a firewall. Typically, academic collaborators only have available to them:

- *Criminologists’ and sociologists’ models of criminal behaviour* that lie within the open domain. These are especially important because they often give a great source through which to both categorise different classes of crime and describe their development.
- *Open-source data about analogous past incidents*. These typically appear in articles by journalists and within a scholarly case studies of specific events written by criminologists. Although these are often not data in a statistical sense, each such report can give information about the development of past instances within a particular category and so inform the CPT of an associated BN.
- *Access to someone from behind the firewall*. Such a person will be free to disclose relevant, less sensitive domain information that might begin to fill out newly arriving information necessary to build both the structure and the probability factors of a probability model with sufficient specificity to be part of a Bayesian decision support system.
- *Securely emulated data* generated through in-house software unknown to the academic co-creator, calibrated with secure inputs. This has proved to be a valuable tool for checking predictive algorithms provided by academic teams—although of course such tests can only be as good as the outputs of the emulation tool used to test it.

It has been established through work in less secure domains [21] that—once the challenges associated with sparsity of data on at least some variables have been acknowledged and addressed—various graphical models provide the ideal framework around which to build well-calibrated models behind firewalls. The sources described by the first and third bullets inform the early structural phases of the modelling processes, whereas the second and third bullets inform the embellishment of the model. Furthermore, the last three bullets enable the model to be tested and refined into a working piece of code which remains securely embedded behind a firewall.

## 3. Criminal Plots

### 3.1. Introduction

Criminal activities can take on a number of guises. Many crimes are simply opportunistic in nature and so are less predictable except in a population sense. But many other crimes—especially serious crimes such as our vehicle attack example—need a degree of planning or preparation. This makes it at least feasible for the police to frustrate the perpetration of individual incidents by appropriately intervening in their preparation. We have argued in [13] that different genres of criminal activities each demand their own type of model. However, we here discuss one particularly interesting broad category of criminal activity that we have called a *plot* [7]. Plots can be described by a subclass of the 2TDBN. So a protocol for establishing a library of plots provides an example of establishing a library of BNs in a context where data are typically missing not at random and are often disguised. Here, because the discussion of terrorist plots is now open source [7,12], our running example will focus on the co-creation of this class of model.

### 3.2. Plots as a Hierarchical Bayesian Model

Plot models—like the ones first discussed in [7]—have been directly elicited from domain experts and can be expressed as a 2TDBN. This BN graphically expresses a 3-level hierarchical model of a plot’s description whose lowest two levels are usually latent, as follows:

1. At the deepest level of this hierarchy is a latent discrete Markov process modelled by a time series of random variables $w^{T}≜\left\{w_{t}:t=1,2,\ldots ,T\right\}$. This discrete time series indicates in which of a number of preparatory phases—elicited from domain experts to characterise a particular class of crime—the given plot might lie at any given time. Represent the phase of a particular plot at time *t* by the indicator variables $\left\{w_{0t},w_{1t},w_{2t},\ldots ,w_{mt}\right\}$, t=1,2,…,T. The particular phase denoted by $w_{0t}$ is called the *inactive* phase—an absorbing state where the criminal has aborted their plot. We call the other phases *active* phases. The time series of an incident’s phase is typically latent to the police—although insider information or occasional revelations might directly inform it.
2. Within a plot, when in a particular phase, a criminal will need to complete certain tasks—characteristic of a certain class of crime—before they can transition to a subsequent phase. At each time step, this intermediate layer of the hierarchical model is a *task vector* $\theta_{t}=\left\{\theta_{1t},\theta_{2t},\ldots ,\theta_{nt}\right\}$ consisting of component tasks. These are often indicator variables on whether the criminal is engaged in the given task or not. Subvectors $\theta_{tI(w_{j})}$, called *task sets*, of the task vector $\theta_{t}$ are defined as those tasks whose marginal distributions for a given active phase $w_{j}$, j=1,2,…,m, are distinct from their distributions for the inactive phase $w_{0}$. So, these activities suggest that the suspect might be in the phase $w_{j}$. Let $\theta^{T}≜\left\{\theta_{t}:t=1,2,\ldots ,T\right\}$. Again, the components of $\theta^{T}$ will typically be latent and only inferred by police, although, on occasion, police might happen to directly observe that a particular task is underway or complete.
3. Typically, the police will have streaming time series of observations called *intensities* $z^{T}≜\left\{z_{t}:t=1,2,\ldots ,T\right\}$ about the progress of a suspected plot routinely available to them. The components $z_{t}=\left\{z_{1t},z_{2t},\ldots ,z_{nt}\right\}$ of the intensity vector are chosen to help police discriminate whether or not an agent is engaging in the task $\theta_{it}$, i=1,2,…,n at time *t*, t=1,2,…,T. We illustrate this below. These are, by definition, seen by police. However, for any ongoing incident, academics outside the firewall will typically not have access to the values of these data streams, at least not until the criminal has been convicted. So often the components of $z^{T}$ and nearly always the values they might take within an ongoing incident will be missing to the academics guiding police support. This information is highly sensitive because criminals could disguise the signals they emitted or even distort these to deceive their observers if they learned what police could see of their activities.

For t=1,2,…,T, the 2TDBN will have as its vertices the components of $\left(\xi_{t-1},\xi_{t}\right)≜\left(w_{t-1}^{t},\theta_{t-1}^{t},z_{t-1}^{t}\right)$, where

$$w_{t-1}^{t}≜\left\{w_{t-1},w_{t}\right\},\theta_{t-1}^{t}≜\left\{\theta_{t-1},\theta_{t}\right\},\;\mathrm{and}\;z_{t-1}^{t}≜\left\{z_{t-1},z_{t}\right\}.$$

Further detail of the factorisations of the joint density of ξ is given in [7], which is consistent with a standard 2TDBN with Markov time-slice graph $G_{t}$, t=1,2,…,T. The full BN $G^{+}$ then simply concatenates these graphs together. Note that, by definition, all vertices of a 2TDBN which are components of $\xi_{t-1}$ are founder vertices—i.e., have no parents—for that time slice.

For plot models, the vertex $w_{t}$ has the single parent vertex $w_{t-1}.$ The subgraph $G_{t\theta }$ of $G_{t}$ generated by the components of $\left(\theta_{t-1},w_{t},\theta_{t}\right)$ is an elicited DAG on the vertices drawn from components of $\left(\theta_{t-1},w_{t},\theta_{t}\right)$ describing the generating latent process. The components of $\left(\theta_{t-1},w_{t}\right)$ in $G_{t\theta }$ are founder vertices. The parents of the components $\theta_{it}$ of $\theta_{t}$ must include $w_{t}$ and all components $\theta_{i^{\prime }t}$$i^{\prime }<i$ where both $\theta_{i^{\prime }t}$ and $\theta_{it},$ for some phase $w_{j}$, are components of the task vector $\theta_{tI(w_{j})}$, j=1,2,…,n, but otherwise any DAG may be valid. This is a generalisation of the model seen in [7] in which they assume independence between these tasks for a given phase.

**Figure 1:**
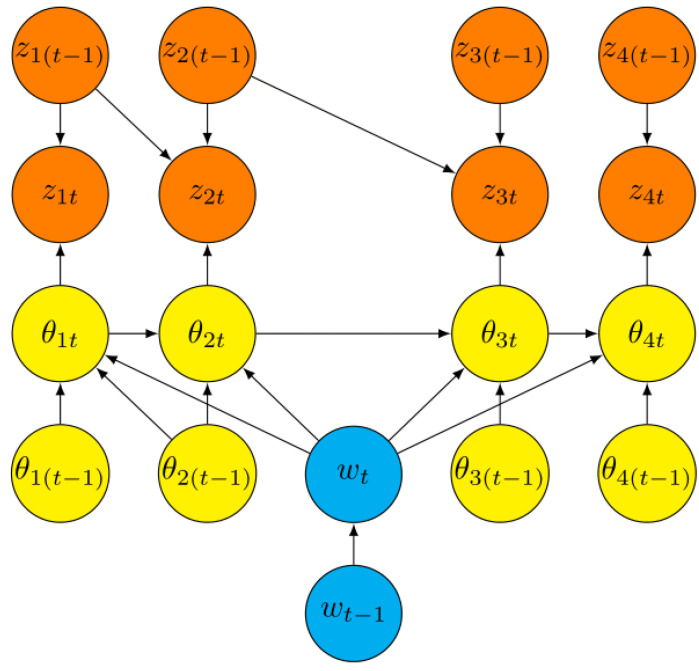
Example 2TDBN for a plot model with four tasks with phases coloured blue, tasks coloured yellow, and intensities coloured orange.

Finally, the subgraph $G_{tz}$ of $G_{t}$ generated by $\left(z_{t-1},\theta_{t},z_{t}\right)$ has as founder vertices components $\left(z_{t-1},\theta_{t}\right)$. For a plot model, we simply demand that, for each i=1,2,…,m, the component $z_{it}$ must have as a parent only $\theta_{it}$ out of the task vector, as well as any of the components of $z_{t-1}$ indexed before it. This is ensured by defining $z_{it}$ so that it directly informs the task $\theta_{it}$ alone, i=1,2,…,m. There are no edges from $w_{t-1}$ or $w_{t}$ to any component of $z_{t}$. This is because the intensities and tasks for plot models are defined in such a way that observations can inform the phase of a plot only through the tasks the criminal engages in to pass through that phase. An example of a graph $G_{t}$ of a 2TDBN valid for all times t=1,2,…,T on the components of $\left(\xi_{t-1},\xi_{t}\right)$ of a simple elicited plot when there are only four tasks for the current phase is given in Figure 1.

We return to the vehicle attack example introduced in Section 2.1. Suppose Figure 1 is modelling the progress of such a vehicle attack. In [7], a similar example of a vehicle attack is presented using synthetic data based on simulations and shareable elicited information. We suppose the current phase $w_{t}$ in Figure 1 is the phase referred to as the ‘Mobilised’ phase in [7]. We see in that paper that this phase has four tasks. Based on this, we suppose that, in our example 2TDBN, we have that $\theta_{1t}$ corresponds to engaging in public threats, $\theta_{2t}$ corresponds to making personal threats, $\theta_{3t}$ corresponds to reconnoitring locations, and $\theta_{4t}$ corresponds to travelling to the location of the attack. Each of the intensities $z_{it}$ are directly informative of the progress of task $\theta_{it}$ and no other task, as stipulated above. We provide a more complex intensity structure than in [7] as we allow an intensity $z_{it}$ to depend on any intensity $z_{i^{\prime }\left(t-1\right)}$, where $i^{\prime }<i$, as well as on $z_{i(t-1)}$. However, we have simplified the structure of the tasks by utilising one-step dependences rather than dependence on all tasks with a lower index. Figure 1 provides an example of how the 2TDBN for one phase of a plot may be structured, and we can verify that our above constraints hold in our example—except for our simplification of the task dependencies. Further numerical analysis of this example can be found in [7].

There are several additional structural properties demanded of a plot model that are not embedded in a 2TDBN. These involve hypotheses about the formation of the task set, the impossibility of passing from some phases to others and the fact that many entries in different CPTs within the subgraph $G_{t\theta }$ must be identical. We are developing a bespoke class of models that more explicitly handles these structural hypotheses and we aim to demonstrate how these models can be efficiently applied to plots in a later paper. However, the more familiar 2TDBN is still a valid framework for inference and is therefore ideal to illustrate the secure elicitation protocol we outline below as it might apply to a BN.

### 3.3. Causal Modelling for Decision Support on Plots

For a plot model, because its variables explicitly describe how and why the crime develops the way it does, it is almost immediate that it is interventionally causal. It describes the precise processes under which an attack might unfold—not just the likely way variables might depend on each other. It therefore transpires that it provides an excellent template for a causal 2TDBN [1]. We also demonstrate that it is consistently causal in the sense that the topology of the 2TDBN of one category will apply to most instances within it, as will many of its CPTs. This is another reason for using a library of plot models as our running example.

However, when the criminal can become aware of at least one of the interventions police might consider adopting, typically the topology of the 2TDBN needs to be more expressive than if this were not the case. If the suspect cannot foresee an intervention from police, they will never deviate from their *modus operandi* (MO), hence no alternatives need to be modelled. If they *can* foresee interventions, they may deviate from their MO—perhaps by considering quieter and therefore less monitored areas for the vehicle attack—and police must consider these deviations in their model. Two different detailed illustrations of how these embellishments can be systematically embedded in a framing graph are given in [13].

### 3.4. Graphs in a Library of Terrorist Plots

#### 3.4.1. Structural Hypotheses of Plots for an Academic Team to Embed

Suppose we are developing a library of graphs that might support the real-time decision-making of police charged with defending the public from various forms of terrorist outrages from a lone attacker such as the vehicle attack described in Section 2.1. We will assume that we are at the initial point of co-creation where we plan the first entry within the library. We provide a little detail of this context below.

Police and criminals are both aware that terrorist plots of the five types mentioned in Section 2.1, including vehicle attacks, typically progress through the generic set of phases described above, all of which need to be completed before any such attack is successful. We note that we have subsequently found that, for the majority of types of attacks in libraries of plots like the ones described above, these phases define the states of the phase variables $\left\{w_{t}:t=1,2,\ldots ,T\right\}$ sufficiently well to provide the predictive capabilities police need. So, in fact, both for a vehicle attack and other attacks above, $\left\{w_{t}:t=1,2,\ldots ,T\right\}$ can be assumed to take one of these six states.

On the basis of open-source data, academic teams have learned that the transition matrices between these six states will, however, be a function of which of the five types of attack the orchestrator chooses, the intent of the perpetrator, their capabilities, and the quality of the defences police might employ. A generic form of the phase transition matrix is provided in Appendix A. For some classes of plot, such as exfiltration plots, some of these phases could occur simultaneously. In this case, we have needed to refine the states in an obvious way so that these are disjointed. However, in terrorist plots, given the categories of type, intent, capability, and defence describing a particular suspect rather than their environment, and especially with the perpetrator acting alone, it is usually safe to assume that the suspect lies in just one of these states at any given time. Henceforth, assume that this is the case for all examples we give here.

Here, we can already make some plausible assumptions about any one of the following five types of plots:

1. At any time, a potential perpetrator may choose or may be forced to abort a plot and thus transitions into the absorbing state $w_{0}$. This means that there is a benefit to the police, making it more difficult for the terrorist to transition through the required phases to be in a position to perpetrate a crime.
2. Clearly, a suspect must have been recruited—phase $w_{1}$—before they can transition to later phases.
3. Once a given suspect is skilled up to be able to perpetrate a plot (e.g., by taking a heavy goods vehicle driving course)—passing through $w_{2}$—within the time frames police would be working in, that suspect will remain trained and will not lose these skills.
4. However, the suspect can review and substitute one identified target location ($w_{3}$) with another, or can acquire a vehicle and then discard it ($w_{4}$) at any time they choose. So, just because a suspect has been in the phases $w_{3}$ or $w_{4}$ at one time does not mean they are currently equipped or have a current target identified.
5. The perpetrator cannot attack—completing phase $w_{5}$—until all other active phases have been completed (and remain so).

So, once the nature of a particular attack has been elicited, many of these structural elements of one model within the library—here concerning how a suspect might transition the phases of the plot—can be immediately shared and translated to other entries in the library. Note that it is not possible to embed these structural hypotheses explicitly within the topology of the graph of a 2TDBN, so these will need to be logged by academics and embedded implicitly within this structure. These structural assumptions can nevertheless greatly simplify the qualitative and quantitative elicitation process.

We often find that some idiosyncratic structural features will often need to be embedded too. For example, the order a suspect chooses to pass through the phases $w_{3}$ and $w_{4}$ will depend on how difficult the equipping phase $w_{4}$ might be. This will typically depend on the nature of the plot as well as the capabilities of the suspect.

In contrast to the largely shared structural expert judgements of police, the collections of tasks associated with different types of terrorist attacks will usually differ significantly across different entries. For example, the tasks undertaken to ensure that a suspect is sufficiently skilled and trained to perpetrate a vehicle, bomb, firearm, or knife attack are quite different from each other, as are the tasks needed to equip themselves for such attacks. Nevertheless, some tasks will often be shared across varying types of plots, especially those associated with communications across co-conspirators and the identification of appropriate targets. This means that some of the entries of CPTs associated with different entries within a library can be shared.

When it comes to intensities, the precise nature of what police might have available to them will often not be known to the academic team. It is in this aspect that the academic stochastic model and the one police use behind their firewall could be very different from one another. We will see, however, that, because of the underlying structure of the plot model, the actual updating algorithms for both models will be identical or very similar.

Furthermore, there are very obvious types of observation that academics can speculate will be available to the police and then build their academic model in light of these speculations. For example, web searches for hiring a heavy goods vehicle are clearly indicative that a suspect is trying to arm themselves for a vehicle attack. So—based on common sense and open-source information about what police *might* have available to them—the academic team can make informed conjectures at least about the broad nature of this genre of information, although possibly not about its reliability and certainly not the values such information might take in any live case.

Some such speculation will be accurate, other speculation less so. Nevertheless, police behind the firewall will be able to weigh the plausibility of any academic speculation and adapt their internal code to correct within their own replica system any poor guesses made by academics. The initial attempts by the academic team therefore guide the police team in modelling the real process by providing a template for how any data might be embedded in a model. The original academic model also demonstrates to the police how valuable certain data streams might be.

#### 3.4.2. Academic Approach to Modelling a Vehicle Attack

Because for this early co-creation the protocol described below was only nascent, describing how we would now proceed becomes hypothetical and will not betray any actual potentially sensitive co-creation between the two teams.

Within this co-creation, the academic team learn that this type of plot typically progresses through a set of phases outlined above for a triaged suspect who has become a person of interest. The academic team are then pointed to the open-source literature which explains that the typical tasks $\theta_{tI(w_{1})}$ a suspect might engage in when in the first active radicalisation phase described above are typically shared with the other plots in the library. These include the individual tightening their relationships with like-minded people and a progressive retreat from day-to-day contact with otherwise close contacts such as certain friends or relatives who might strongly disapprove of the plan. Academics therefore learn from open sources that by monitoring the suspect’s web activities and social media, through phone logs and through learning about recent interactions with known IS sympathisers using CCTV, that the intensity of such activities will inform police about the likelihood that the given suspect is within this phase, or was in this phase at some time in the past. Therefore, they can conjecture that measurements of these might be the tell-tale signs (intensities) police might use as subvectors of components $z_{I(w_{1})}^{T}$ of $z^{T}$. Although police capability to harvest such information within suspected incidents will be highly confidential, the academic teams can nevertheless create for themselves synthetic data streams about various hypothetical incidents and capabilities that can demonstrate how the statistical model might inform the police about whether and when this phase was enacted. Early illustrations of this process appear, for example, in [7] and elsewhere in police handbooks.

To realise the plot, a suspect who cannot yet drive a heavy goods vehicle would then need to somehow increase their capabilities to learn how to do this, denoted as phase $w_{2}$. Associated tasks in the middle layer of the hierarchy might be to sign up for a heavy goods vehicle training course or to learn this skill from an accomplice—perhaps located abroad—already able to drive and who has access to such a vehicle. All such options at time *t*—denoted by the task subvector $\theta_{tI(w_{2})}$—are again common sense and so are also obviously available to the academic team. Note that if police have information that the suspect already has this training then of course they pass instantaneously through this phase. Their code can obviously be designed to facilitate the accommodation of such very incident-specific information.

The academic team could also conjecture plausible signature intensity measures of such activities available to the police team; for example, discovering the suspect has searched for a commercial driving course or finding evidence the suspect has attended such a training course. Alternatively, police might observe searches for travel or booking of flights to a country where they could receive such training, or might observe association with a local friend known to have these skills and capabilities. Other information might be more physical; for example, from CCTV images to observe the suspect at an airport or driving such a vehicle with a friend.

The suspect will then need to *identify a target* and reconnoitre it—phase $w_{3}$—where he could cause the most drama and spread the most fear, individually or working with accomplices. The tasks associated with this phase are typically common to all types of attack and have appeared in press reports and scholarly articles and so again are largely known to the academic team. These include investigating the demographics and defences of different candidate sites which could involve visiting potential target sites or electronically exploring maps of potential target areas. These two possible tasks are represented in $\theta_{tI(w_{3})}$. Again, the academic team might conjecture that such activities might be captured by police monitoring the suspect’s web data, their interception of metadata associated with phone messages, CCTV, and direct observation, which could all form arguments in the intensity function $z_{I(w_{3})}^{T}.$

Once sufficiently trained to perpetrate the plan, the suspect will also need to *source the heavy goods vehicle*—phase $w_{4}$—to use in the attack. This phase will typically involve either hiring such a vehicle, being given it, or stealing it. Again the academic team could speculate the tell-tale sign police might be able to observe. Finally, they will then need to drive this vehicle to the target to perpetrate the attack—phase $w_{5}$. This action could be observed in obvious ways from, for example, traffic cameras, direct pursuit, and intercepted phone messages. Academics can then build models that harness and then customise state-of-the-art feature-extraction algorithms to construct putative inferential methods that can be demonstrated on synthetic data and real open-source data from more benign applications. Police can then use these analyses to template their own algorithms for use within their own secured system.

The point of discussing the above is to demonstrate how, in a given instance, the academic team can build up a structural model that is close to a faithful representation of reality. They can then code up a statistical model of this type of plot that is very similar to one the police team might like to implement. What remains conjectural is structural information about the nature of the intensities possibly available to the police and information they might have about that particular person—for instance, whether or not they were trained to drive a heavy goods vehicle—to model a particular suspect within a particular potential incident. But such information would anyway need to be customised to the particular incident by police behind their firewall.

Of course, the relevant CPTs would need to be constructed to demonstrate the 2TDBN. But even then, note that many of the quantifications needed for such a model—for example, how quickly the suspect could reach a target when driving their acquired vehicle—can be elicited directly by the academic team based on open-source data, or otherwise plausibly guessed.

On the basis of such structural information, academics can build and demonstrate a plot model of such an attack [7]. Obviously analogous Bayesian structural models of other types of terrorist plot attacks listed above could be constructed by the academic team. Such models can then be donated to the police to help guide the construction of their own more realistic in-house matrices and algorithms that can be used to support an actual secure decision analysis to help defend a threatened vehicle attack.

In the next section, we provide, for the first time, a detailed description of how such a library of 2TDBNs can be securely transferred from an academic team to an operational police model despite significant amounts of the in-house data being missing to the guiding academic team.

## 4. Co-Creating a Library of Plots

### 4.1. Introduction

Broadly, the constraints for a secure technological transfer of any class of plots are experienced as follows.

1. A generic description of plots expressed in the *phase relationships* in the lowest layer of this hierarchical model lies largely in the public domain—within sociological and criminological articles, open source case studies and information that police experts can freely communicate. So, this is directly accessible to the academic co-creators who can guide its accommodation into this structure. These helpfully categorise and explain various plots and the motivation and capabilities of various different types of potential perpetrators of the plots in focus. Some generic evidence to inform the generic priors on the CPTs associated with the transition between stages will also be available. This can later be refined by police using more secure information available only to them.
2. Generic information about the *tasks* that need to be completed to move from one phase to another, and the probabilities linked to both the choice of the task and the ease of completion associated with various categories of criminals, is also straightforward for the academics to elicit from sources mentioned in the bullet above. Again—using instructions from the academic teams—the CPTs can then be refined by police using other more sensitive evidence they have about various crimes, using as priors the probabilities on the CPTs based on open-source data.
3. On the other hand, the full extent of the *data streams* police might currently have available to them indicating which tasks are currently being engaged in *within any ongoing investigation* can be highly confidential. For example, if a criminal learned that their messages on the dark web can be unencrypted, then they can use this to disguise their messages or deceive police and thus be harder to apprehend. However, independent of police and based on open-source data, the academic team can of course conjecture what these might be, as we illustrated above. They can then communicate this open-source model to the police, populating the topology of the BN and the associated CPTs ready for adjustment of the police model behind the firewall in light of what only they know.
4. The *actual data* that police collect *concerning specific individuals* are highly sensitive and cannot be shared with the academic team. Were the current suspect to learn what the police knew about the progress of any plot truly in progress, they would become far more dangerous. On the other hand, any personal information about a given suspect cannot be shared until they have been convicted. If the suspect is in fact innocent, then, as soon as this has been discovered, any personal information about them cannot be ethically retained. Such information might be about the category assigned by police to a suspect, the nature of the information being collected on them, and the values of this data at any given time. However, there is a rich, although usually still incomplete, bank of open-source information about proven criminals, for example, provided by press releases and court reports. So, based on academic conjectures and rehashing particular use cases, the academic team can demonstrate how the model might learn in light of such synthetic datasets and share this with the police. The potential usefulness of the open-source code can then be demonstrated as documented in [7].

With these points in mind, we next briefly outline the protocol we have used in co-creation projects with various policing organisations, not just for terrorist plots of the kind above, but to build other libraries of plots. We note that the development of the libraries we discuss below can be incremental—gradually adding and refining entries in the library to progressively increase the scope of the decision-support tools made available to the police. Even partially populated libraries can be extremely useful to the police, especially if the established entries are models for commonly occurring categories of crime.

### 4.2. A Protocol for Co-Creating Plot Models

#### 4.2.1. Notation and Setting

Here, we describe a generic protocol that reflects our current processes, and that enables an academic team to co-create libraries of BNs where police are led to their own Bayesian model to support their SEU decision-making when pursuing a criminal in a way that all information that police need to keep secure remains undivulged. We first need to set up some notation.

Any particular incident whose progress is being monitored will involve a certain broad category of suspect (e.g., an IS sympathiser, their age and their history) which will inform the nature and speed of their progress through the different phases of a plot through the suspect’s intent, potential capabilities, and MO. We denote this background information by ${𝒮}_{b}$. The academic team will be able to discover from open-source publications how police may be able to categorise a given suspect, although the nature of such information may be covert and known only to the police. A second classifier effectively concerns the environment ${𝒮}_{e}$ in which the suspect operates, such as place of residence, which might affect the capability and ease of aborting a plot—information that is more easily exchanged across the firewall. Let $𝒮≜\left({𝒮}_{b},{𝒮}_{e}\right)$ denote this information. We expect that all CPTs will need to be indexed by 𝒮, although most of these will be shared across categories 𝒮. Because the academic team will only need to *demonstrate* their code within their own library, they typically need to only code up one such category. For this reason, we have suppressed this indexing in the development below. However, in the parallel library developed by the police, they will need to elicit different entries for each CPT for each such category.

Denote by $L=\left\{M_{1},M_{2},\ldots ,M_{k}\right\}$ an arbitrary library of fully embellished probability models—called *entries*—where these entries have been ordered consistently with their arrival in the library. Here, $M_{i}=\left(G_{i},C_{i}\right)$, where $G_{i}$ denotes the DAG of the BN and $C_{i}=\left\{C_{vi}:v\in V\left(G_{i}\right)\right\}$—with $V(G_{i})$ the vertex set of $G_{i}$—the collection of CPTs needed to embellish $G_{i}$ into a full probability model, i=1,2,…,k. In [13], by defining an appropriate causal algebra, we demonstrate how the academic team can design the graphs $G_{i}$ so that these provide a valid framework to describe not only how events might unfold not only when police do not intervene (decision $d_{\phi }\in D$) but also when they do (decision $d\neq d_{\phi }$, d∈D). So, in this sense, $G_{i}$ will provide a suitable framework to describe the structure of *any* probability model for all contemplated interventions and categories of suspect–environment pair 𝒮.

Despite this useful structural invariance, in order to build a full probability model for each intervention, police will need to also specify for each category 𝒮 the CPTs

$$C_{di}=\left\{C_{dvi}:v\in V\left(G_{i}\right)\right\}$$

associated with such decisions $d\neq d_{\phi }\in D$, where we write $C_{vi}=C_{d_{\phi }vi}$. Henceforth, we focus on building the library for a fixed category 𝒮. To establish their library, police will then need to repeat the process below for all other categories of suspect 𝒮.

It is convenient to partition the sets $\left\{C_{di}:d\in D\right\}$ of CPTs into the three sets $\left\{C_{di}\left(1\right),C_{di}\left(2\right),C_{di}\left(3\right):d\in D\right\}$, where $C_{di}\left(1\right)$ denotes those CPTs whose prior information and informative datasets can be shared with the academic team; $C_{di}\left(2\right)$ those CPTs for which the academic team have some information and perhaps data informing these but for which police have additional information; and $C_{di}\left(3\right)$ those CPTs for which the academic team only have scant information and which police would overwrite with their own secret but much more reliable information. Let $\left\{C_{di}^{\prime }\left(1\right),C_{di}^{\prime }\left(2\right),C_{di}^{\prime }\left(3\right):d\in D\right\}$ denote those sets of CPTs for a new entry which have been elicited as different from any yet to appear in the library—i.e., for j∈{1,2,3}, with i=1,2,…,k indexing the models in the library, define

$$C_{di}^{\prime }\left(j\right)≜C_{di}\left(j\right)\setminus \left(\cup_{1\leq i^{\prime }<i}C_{di^{\prime }}^{\prime }\left(j\right)\right)$$

Note that, for this construction to make sense, we assume we have labelled the vertices in $V(G_{i}),i=1,2,\ldots ,k$, such that vertices with the same index have the same meaning across different entries in the library—here across different criminal plots. In the protocol described below, academics will need to craft the naming of vertices so that these are as generic as possible so as to make the association across different entries in the libraries as fluid as they can be. We note that it is often necessary to revisit a generic naming of vertices so that the meaning of the vertices continues to apply to all entries (see, e.g., [11]). We also assume that this will mean that vertices, if they appear in two different graphs, will be ordered compatibly with each other. Thirdly, in the protocol defined below, we will assume that the graphs describing the criminal missions—here plots—have been chosen by academics so that these will be causal in the senses we have discussed above. This will mean that, for each category of crime, the graphs of the progress of the criminal mission will be respected before and after any intervention police might contemplate.

Because the libraries we construct have entries that describe similar crimes, logic often demands, or it is at least plausible to assume, that the dependence structures they express through the topology of the graphs within a library are shared. We have also argued that some of their CPTs will also be shared. It is therefore helpful to introduce a notation which can reflect these commonalities over the *k* models already located within the library. So, let $G_{*}≜\cap_{i=1}^{k}G_{i}$ denote the graph with vertex set $V\left(G_{*}\right)≜\cap_{i=1}^{k}V\left(G_{i}\right)$ with a directed edge $\left(v^{\prime },v\right)$ from $v^{\prime }$ to *v* in the edge set $V(G_{*})$ if and only if the edge $\left(v^{\prime },v\right)$ lies in at least one of the edge sets $E(G_{i})$, i∈{1,2,...,k}. Similarly, where $V_{*}\subseteq V\left(G_{*}\right)$, let $C_{d*}≜\left\{C_{dv*}:v\in V_{*}\right\}$ for some d∈D denote the set of those CPTs that are shared by all graphs in the library $G_{i},i\in \left\{1,2,\ldots ,k\right\}$. Note that a necessary condition for $v\in V_{*}$ is that *v* has the same parents in each $G_{i},i\in \left\{1,2,\ldots ,k\right\}$, so that all CPTs have the same dimensions. This notation sets up a way to find these common CPTs in a large library to aid the construction of priors for a new library entry.

Before the construction of the library begins, the academic co-creators train at least one of the police team so that they are able to elicit from colleagues the probabilities that might be needed for CPTs whose values must remain behind the firewall. This would typically encourage them to sign up for one of several open courses in probabilistic elicitation methods. Some of these methods include variations of the Delphi method (see, e.g., [22]), Cooke’s classical method [8], and the IDEA protocol [23], all of which involve asking groups of experts for their probabilistic judgements and evaluating these judgements over a number of stages. These tend to rely on the mathematical aggregation of experts’ judgements, as a consensus is not usually naturally reached among the experts. However, an expert panel formed of members of police teams who are accustomed to working together are more likely to reach a consensus about probabilistic judgements surrounding an ongoing criminal plot. Therefore, elicitation methods focused on behavioural aggregation may be favoured over the aforementioned methods. The main elicitation technique of this kind is the Sheffield Elicitation Framework (SHELF) [24], in which a facilitator guides the sharing of information and leads group discussions with the aim of reaching a consensus among experts. More detail on these elicitation methods can be seen in [9,25]. Members of the in-house police team now trained in such an elicitation method would then receive more customised training via the academic co-creation team. Such activities might involve the academic team engaging them in elicitations of the CPTs within the first iteration of the academic library then donated to the police as a template of the model behind the firewall.

Denote the library built up by academics outside the firewall (police behind the firewall) on iteration *t* of its development by $L_{t}^{A}$$\left(L_{t}^{B}\right)$, and use the same labelling convention for all entries and their pairs within these libraries. We write the existing entries in the libraries as $\left\{M_{1},M_{2},\ldots ,M_{k-1}\right\}$, but these libraries may be empty. We are now able to describe a protocol for co-creating the next entry $M_{k}$ into this library.

#### 4.2.2. Step 1—Initial Library Based on Open-Source Information

The academic team first set up the initial prototype version of their expanded library $L_{1}^{A}$ as follows:

1. Informed by previous studies undertaken when building $\left\{M_{1}^{A},M_{2}^{A},\ldots ,M_{k-1}^{A}\right\}$ within the current academic library $L_{0}^{A}$, supplemented by other open-source information pertinent to $M_{k}^{A}$, and guided by police sharing their own open-source knowledge, academics choose a graph $G_{k}$ representing the BN of the next category and type of crime. This stage typically involves further engagement with experts and a deep dive into the literature to discover new information about the new entry to the library.
2. The names of the vertices in $V(G_{k}^{A})$ are made as compatible as possible with the names given to vertices in $V(G_{i}^{A})$, i=1,2,…,k−1. Note that this sometimes entails the relabelling of the vertices in this set in light of the meaning of vertices in the new vertex set $V(G_{k}^{A})$. This is a delicate process (see, e.g., [11]), albeit in the very different context of ecological modelling. This harmonisation step helps to maximise the amount of local structure and probabilistic information that can be shared across different BNs in the library and so helps minimise duplicating effort establishing the next entry in the library.
3. We next begin populating the CPTs as these apply to the new entry of the library which would be valid were police not to intervene. The academic team first elicit from police the likely nature of the partition of the CPTs $\left\{C_{k}^{A}\left(1\right),C_{k}^{A}\left(2\right),C_{k}^{A}\left(3\right)\right\}$. They then need to elicit from the police team which of these CPTs—because of their shared meaning with other models in the library—already appear in the library, and which CPTs $\left\{C_{k}^{\prime A}\left(1\right),C_{k}^{\prime A}\left(2\right),C_{k}^{\prime A}\left(3\right)\right\}$ are unique and hence need to be populated.
4. To populate $C_{k}^{\prime A}\left(1\right)$ and $C_{k}^{\prime A}\left(2\right)$, the academic team then proceed as they would in non-secure settings. They first elicit the uncertain probabilities in $C_{k}^{\prime A}\left(1\right)$. They then refine these judgements based on available data using the Bayes Rule to construct open-source posterior tables. They repeat this process for CPTs in $C_{k}^{A}\left(2\right)$. At this stage the academic team can use their skills to identify and adapt statistical and AI methodologies, in particular to use time series data extracted from particular past instances to calibrate and efficiently estimate the parameters associated with these probability tables.
5. Dummy entries are then chosen by the academic team for those new CPTs in $C_{i}^{\prime A}\left(3\right)$ that will become informed primarily through secure in-house information, and labelled as such. We recommend that these dummy entries are chosen to appear as plausible as possible to the police and, where possible, calibrated against available open-source case studies—at least as they might apply to one category of suspect 𝒮.
6. We note that CPTs may need to be populated uniquely for different types of suspect 𝒮. The MO and training of a right-wing terrorist recruit will be different from a terrorist affiliated to IS which in turn will be different from a suspect acting completely autonomously. In our running example, it will usually be possible for police to reliably categorise any given triaged suspect, although, in some plot libraries—like those designed to protect against exfiltration attacks—such prior categorisation will be less certain. In either case, when the library is designed to be applied to make inferences about different categories of criminal, different collections of CPTs will need to be constructed for each such category. However, we note that most CPTs will be shared across different categories, but those that *do* differ help to formally discriminate the possible type of suspect faced by police when this is uncertain.
7. Academics next need to populate their CPTs $\left\{C_{di}:d\neq d_{\phi }\in D\right\}$ associated with each potential intervention they contemplate making. To do this, they repeat the elicitation process described for the unintervened process in the three bullets above for different categories of suspect 𝒮. Superficially, this might look to be a very large task. However, if the BN is well-chosen, whenever it can be described as causal, as we have argued above that plot models can be, this will typically only require the addition of a few select CPTs for each potential intervention. This will be so, even if it will be apparent to the criminal that such interventions have been made.
8. The models are then coded up as software. The code developed by the academic team will be much larger than the distilled code that will be delivered to the police team in order for the academic team to be able to explore various modelling choices that the police team do not need to do themselves, as well as to allow rigorous verification to be performed before the library is transferred to the police. The academic team check the plausibility of the outputs of this code and the faithfulness of the code itself against synthetic cases. The academic team first simulate use cases from their model. They then use open-source data about real past incidents, supplementing these with any synthetic data about records they believe might have been observed but are now lost, performing the standard statistical diagnostics normally used to check the performance of this BN.
9. As part of their development, the academic team will have embedded a suite of estimation modules and diagnostic checks for the new model using these data for police to later replicate. Examples illustrating this process can be found in [7,12].
10. The rationale behind both the chosen structures within the library, the real CPT, any data used to calibrate these and the methodology to accommodate them are all recorded for future appraisal by police. It is vital to carefully provide the in-house team with a report carefully documenting the rationale behind the choice of model for the new category and to add this to any other such documentation as this applies to previous models in the library. For an example of the description of the outputs of such software and their embedded algorithms, see [7,12].
11. Because these statistical models are all based on open-source information, they can be freely submitted to proper peer review and criticism. The models, methodologies, and applications can thus be properly quality controlled to this point for later technical revision if this is necessary at the earliest stage of the co-creation.
12. A handbook is created or modified for the new library. This includes how the new well-documented transferred code works. It also demonstrates the estimation, statistical diagnostics, and dummy examples provided which in-house statisticians are then able to replicate for the recent library entry. The handbook and a distillation of the code—both based solely on all open-source materials—is then delivered to the police team.

#### 4.2.3. Step 2—Police Team Create Their First In-House Library

At this point, the police team will have received the latest version of the academic library $L_{1}^{A}$, including the model of the newest crime entry for a selection of categories 𝒮. They will also have received a distillation of the code used by the academic team in building and verifying their models. The code shared with the police team will be heavily simplified and will only feature critical components for police to replicate the chosen methodology of the academic team. They now need to use this code and academic library to help develop their parallel library $L_{1}^{B}$, informed by their current library $L_{0}^{B}$ of coded models located behind the firewall, to add the new entry into their own library. This will involve refining $M_{k}^{A}$ with the enhanced data available only to them to produce a new entry $M_{k}^{B}$ for their own library. They will have the capability to run any of the coarse models in the latest academic library $L_{1}^{A}$ as well as the more refined coded models in the latest police library $L_{1}^{B}$. We recommend they translate the latest crime model $M_{k}^{A}$ in the following way:

1. Police take the delivered code and the enhanced library $L_{1}^{A}$ and test that they can run and emulate the results provided in the handbook of the delivered system. This ensures that the new library $L_{1}^{A}$ has been successfully transferred.
2. The performance of $M_{k}^{A}$ within $L_{1}^{A}$ is then applied by both co-creation teams to any available securely emulated data carefully constructed and delivered by the police team. This is conducted through calibration using secure information to provide the academic team with shareable, informative outputs. This can be used to check whether or not the outputs of the academic model look broadly plausible to the police, given their model is only informed by open-source data. If this is not the case, then the academic team need to liaise with the police team to adjust $M_{k}^{A}$. However, within this co-creation step, it is important for both teams to bear in mind that this quality-control step will only be as good as the in-house emulated datasets.
3. Conditional on this emulation being verified, the mismatch is likely due to either the misspecification of the elicited graph $G_{k}^{A}$ or the inaccuracy of the academic guesses about the secure CPTs. In the former case, the academic team will need to perform further elicitation to resolve this issue, as they would in contexts where there is no security issue. In the latter case, the police team will need to give hints about how the priors within the open-source model might be better calibrated to reality, or, if this information is too sensitive, to acknowledge the discrepancy and nevertheless retain the mismatching entry.
4. The next step is to translate the extended library $L_{1}^{A}$ containing the new entry $M_{k}$, taking the current police library $L_{0}^{B}$ behind the firewall and adding an adjusted version of this model to form an initial construction of $L_{1}^{B}$. Note that any non-empty extant police library $L_{0}^{B}$ will typically contain a more refined suite of models than the entries in the library $L_{0}^{A}$ used by academics. In particular, the CPTs in $C_{i}^{B}\left(2\right)$ and $C_{i}^{B}\left(3\right),i\in \left\{1,2,\ldots ,k-1\right\}$, may be much more accurate than their equivalents in $L_{1}^{A}$. This, in turn, will mean that the CPTs for $M_{k}$ matched to other models in the library should give more reliable results when applying $L_{1}^{B}$ than $L_{1}^{A}$. This will need to be acknowledged within this translation step.
5. Police then adjust the pre-existing BNs within this initial construction of $L_{1}^{B}$—such as changes to the node names to more generic terms so that these will be consistent with the revised library. They then make any necessary adjustments to the topology of $G_{k}^{A}$ to contain any structural information known only to them. In the case of plot models, these additions are most likely those associated with intensity measures they might secretly use to inform them about whether or not various tasks are being undertaken by a suspect. This is because academic teams are more likely to make erroneous guesses about the highly secure information police have that form the intensity structure of the plot model than they are about the task and phase structures of the plot model. Any new types of undisclosable measurements they might have available that relate to tasks in $M_{k}$ but to no other entry in the library will need to be represented.
6. Police have been trained to elicit in-house any prior probabilities needed for the secure CPTs $\left\{C_{di}:d\in D\right\}$. This is the most delicate part of the operation to manage. It is useful for any in-house representative who has not been trained in probabilistic elicitation and who will need to act as a facilitator to first attend one of the currently available aforementioned probability-elicitation programmes. We have also found that the in-house technician can often benefit from more bespoke training delivered by the expert academic team, where they are part of a mock elicitation, as directly as possible.The academic team will be ready to answer any generic questions the in-house facilitator might have about this process. This will need to be repeated for all categories of suspect–environment pair 𝒮.
7. Police then populate the prior probabilities needed for the secure CPTs elicited and facilitated by the trained in-house representative behind closed doors. These CPTs will usually consist of analogues in $C_{k}^{B}\left(3\right)$ to the CPTs in $C_{k}^{A}\left(3\right)$—where academic guesses of the topological structure of the network are accurate—as well as the CPTs associated with any new intensity measures that the academic team did not include in their model based on open-source information.
8. Data are then embedded by the in-house expert, emulating how they have seen the academic team do this for CPTs whose expert judgements are not sensitive and whose training data are open source. Because the mathematical equations and supporting code for performing these tasks tend to be generic, such information can usually flow freely between the two teams.
9. Police will now be able to use their adjusted code and algorithms to make their improved predictions and inferences. They then emulate the statistical estimation and diagnostics they have seen the academic team apply to calibrate their models and check their plausibility against the totality of data they have available to them. If through this process they discover inadequacies in their model, they share these with the academic team—see Step 3 below.
10. Otherwise, they write their own in-house handbook to the new code using the handbook of open-source software as a template to support other in-house users and to prepare for the next library entry.

#### 4.2.4. Step 3—Police and Academics Toggle Models Until Requisite

It is unlikely that this first version of the in-house library $L_{1}^{B}$ is totally calibrated and well-estimated. Our experience has been that there will be latent problems identified by police concerning the validity and plausibility of the inferences the code might generate. Problems can often include the code not running at all or being extremely slow, the outputs becoming absurd, and the inferences being too uncertain to provide any useful level of decision support. However, the fact that $L_{1}^{B}$ was seeded by $L_{1}^{A}$ means that the academic team are improving their current library so that it is fit for purpose.

The next step is to help the in-house team produce code which appears at least superficially to produce plausible outputs. Because the academics understand the functionality of the methodology, algorithms, and code that seeded it well, they are much better informed than they might otherwise be to help the in-house team address these problems at arm’s length.

1. For any identified problems about the outputs of their internal code, police feed back carefully sanitised points of concern about the code or outputs of their in-house models associated with $M_{k}^{B}$ to academics. Examples might be “$M_{k}^{B}$ seems to consistently underestimate threats of people of type x”, “The vast amount of data we have available means that estimating various parameters of the model using the analogues in $L_{1}^{A}$ simply does not seem to work”, “We have no data at all that might inform x given y, and no obvious source of expert judgement either”, and “For realistically informed secure use cases, the system seems to learn far too slowly to provide operational decision support”, just to give a few.
2. Vigorous conversations now take place between the two teams about the problems communicated in a sufficiently generic way to keep all confidential information behind the firewall. The types of alterations the academic team might perform may include changing the topology of the graph, the parameters, the probabilities, and the estimation algorithms in $L_{1}^{A}$ for police to copy in modifying entries in $L_{1}^{B}$. Examples of replies from academics might be “It appears we might have missed within our description of the underlying processes some of the ways that these threats might happen—perhaps we can find these?”, “Method y has proved to be a very efficient feature extraction algorithm in analogous settings. We provide a version of this that you might be able to embed in your code”, “Do you have effect datasets about past incidents whose outputs might allow these explanatory parameters to be calibrated? If so we will show you how to use this to set plausible values of these probabilities”, and “We suspect that you have set the signal-to-noise ratios too high—if you set it in this way then...”, among others.
3. This co-creation step continues to be iterated until the model is requisite, i.e., there are no remaining shortcomings the in-house team can find that make the outputs of their in-house version appear inadequate [2,26].
4. The in-house team check the performance of their model on confidential datasets about past criminals or current suspects. Any new issues like the above are then shared securely with the academic team to help find solutions. Through this interaction, the teams make all necessary adjustments and co-create the latest versions of the libraries we shall denote by $L_{2}^{A}$ and $L_{2}^{B}.$
5. The in-house handbooks of these two libraries are rewritten and annotated, templated on the previous handbooks, embedding all the new structures and settings from this last interaction. In particular, this should include the rationale for choosing its settings and any new synthetic and real examples appropriate to each given library.
6. The academic library is now ready for the input of the next plot model so that the co-creation development cycle described above can be repeated as necessary.

#### 4.2.5. Maintenance of the Library as Environments Change

Once an entry within the in-house library is functioning appropriately, it is likely to need to be maintained. This is because criminal processes and the ways they are most likely to be perpetrated evolve. However, because of the way these models have been structured, once a graphical model has been set up, it is relatively easy to maintain. So, for example, for typical plot models, the phases a criminal needs to traverse and the speed they can do this usually do not evolve quickly and these task probabilities only change at a moderate pace. The type of tell-tale signs measured by intensities do change quickly, of course, both in light of technological refinements and the evolving MO of various potential criminals. But these issues can be addressed simply through regular in-house refreshing of the CPTs and occasional small topological modifications of the BN, and police will have in-house specialists to facilitate this. Again, because the academic team have seeded the code, they can actively engage in this maintenance, providing new inputs to any open-source components of the code and methodological advice about how to refresh all parts of the model.

Such maintenance is vital to perform regularly but is relatively resource cheap to the police once the initial system is in place. Again, because of the parallel development, much of the necessary updating can be delegated to the academic team and those elements that need to be securely protected can be routinely updated in-house, guided by the well-informed academic team.

## 5. Uncertainty Handling in a Secure Library

### 5.1. Introduction

In this section, we describe how to address uncertainty handling when following this protocol, acknowledging that many of the data streams that might inform this process are incomplete. We will focus mainly on those aspects of uncertainty management that are central to this secure context.

We begin by outlining how three types of additional structural information about plot models greatly simplify the population of the CPTs of the BN. We then review methodologies translated from extant sources associated with learning within the more general class of Integrating Decision Support Systems (IDSSs), for which the 2TDBN we use here is a special case, when such CPTs are uncertain. This enables us to use the sporadic data police have available to both refine prior settings for a given instance and embed real-time inference about a given suspect as their actions unfold. In particular, we discuss the use of global priors which keep the process description modular and greatly aid inference across the whole library.

We then discuss how a prior for a particular model can be set up, and describe how, once set up, a model drawn from a library of plot models can be applied to a particular individual suspected of being engaged in a particular plot featured in the library. We discuss how the Bayesian paradigm can be used to formally embed various different types of incomplete and indirectly informative data sources that might be available to the police. We end by discussing how these methods can also be applied by police to generate probabilities for the likely impacts of various interventions that they might consider making.

#### Three Special Structural Features of BNs of Plots

There exists extra implicit structural information that simplifies the specification of the CPTs of the 2TDBN of plot models. The first is the explicit assumption of a pattern of zeroes within the transition matrix for $w_{t}|w_{t-1}$ of the phases capturing police knowledge that it is impossible for the criminal to transition from one phase to particular other phases in a single time step. For example, by definition, it will not be possible to transition from phase $w_{0}$ to any other phase or, under the usual choice of time index, for someone to transition from $w_{1}$ (being recruited) to $w_{5}$ (travelling to the scene of the attack). This means that there are far fewer probabilities needing to be elicited to specify the CPTs than for a more saturated model (see Appendix A).

Second, for plot models, we assume that, in the active phase, the distribution of tasks and their relationship structure can be represented by a given BN independent of the phase of the plot the suspect is in [7]. Of course, the conditional distributions of the tasks for each active phase, as well as for the inactive phase, will be distinct. Indeed, these differences will help determine whether the suspect is engaged in the plot and, if so, which phase they are likely to have reached. This therefore justifies police focusing on particular critical subsets of tasks to discriminate the current phase of the attack. We give an explicit representation of this in the next section.

Finally, task sets define those tasks whose CPT entries for a given active phase are different from those conditional on the inactive phase $w_{0}$. For a given phase of a plot, many tasks are not critical, or indeed useful, for the transition of a suspect to a subsequent phase [7]. Therefore, many task CPTs for each active phase are simply duplicates of the task CPTs appearing for the inactive phase $w_{0}$, greatly simplifying the elicitation and inference task.

### 5.2. Setting Up Uncertain Priors Within a Library

#### 5.2.1. Introduction

Within a library of plot models, it is necessary to set up prior distributions for each particular incident, mainly through prior estimates of CPT entries. These will be informed by past incidents, some of whose developments will be exchangeable with the unfolding case. The interesting challenge is whether it is possible to apply standard formal Bayesian methodology to the different library entries in a modular way, and whether the distributions of the CPTs of the model within the library can be updated so that, posterior to learning from similar past incidents and other sources, the distributions remain modular.

The surprising answer to this question is ‘yes’; the necessary theory and conditions for this property were established long ago for the BN (see, e.g., [27]) and in fact remain true even when applied to models with other graphical structures (see, e.g., [28]). These earlier developments were designed for single models, but they transfer straightforwardly and seamlessly on to inferences within libraries in ways we outline below.

In this section, we first review the conditions for when a single BN can be updated such that the posterior distributions of each of its contributing CPTs can independently be functionally updated. We then apply this result to develop analogous results about libraries of such models where different collections of models and CPTs are assumed exchangeable across the library. For the purposes of this paper, we will focus only on this modular learning as this applies to the BN, although analogous results apply to libraries of other types of graphical models too.

#### 5.2.2. Modular Learning on a Single BN

The assumption that the matrices of prior entries across the different CPTs of a BN are mutually independent is called *global independence* [2,27]. There are always globally independent priors available for any BN because—unlike some other graphical models—its CPT entries are functionally independent. Although this assumption can be broken, for example, when a facilitator believes that an expert from whom the conditional probabilities elicited from different CPTs may exhibit the same biases—most routine choices of prior which express only vague judgements would automatically satisfy this condition. When, as in our running example, all the CPTs of the 2TDBNs in our library of models are finite and discrete, one obvious choice is to give all the rows of each CPT independent Dirichlet distributions or independent logistic distributions. These would give two examples of globally independent priors on the entries of the model.

This assumption is so useful as the property of global independence continues to hold posterior to data being used to refine the probabilistic expert judgements within each of the different CPTs of the BN. There are two such types of data that preserve this property:

1. *Ancestral data* from given past incidents. If, after such events, the values of all the variables can be reconstructed up to a given point, so that if data are available for a particular variable then it is also available for all its parents, then global independence will automatically hold a posteriori. This means we can simply perform a prior-to-posterior analysis on each of the CPTs—using relevant count data from criminals advancing through the phases of their plots.
2. *Designed random sampling data* from relevant observational studies. Here, we choose to sample from various CPTs in the model—assumed to be invariant across incidents—stratifying the sample across the different combinations of the levels within the CPTs. When considering a library of plot models, we would recommend sampling from the task CPTs conditioning on the inactive phase $w_{0}$. Note that such sampling data will usually be open source being informed by actions of innocent people and thus shared across the libraries of both the academic and in-house team. Some of the task-dependence data associated with criminal activities may only be available to the in-house team, however, and so can only be accommodated in this way in-house.

The reason why global independence continues to hold in such settings is that, in both cases, the likelihood on the parameters in the different CPTs *separates*; for a formal definition of this, see, e.g., [2]. It means, for example, that it is simple for the in-house team to update their priors using the secure information available only to them in addition to the open-source data available to the academic team. Both can then use a simple Dirichlet conjugate prior-to-posterior analysis whose relevant formulae the academic team can relay across the firewall, as well as simple exponential waiting times for CPTs when the underlying phase transition process is assumed to be a semi-Markov process [29]. In this setting, the in-house model will mirror the model from the academic team, but will feature different values for its hyperparameters. There is now vast literature and training courses available that the academic team can use to enable police to do this.

On the other hand, when we need to formally update models with data that are not ancestral or designed—often data suffering from missingness—typically global independence posterior to such data no longer holds. Indeed, inferences from such data can be very subtle and the signals it can give are often ambiguous. For some examples of the sorts of ambiguity of interpretation that can occur in even the simplest BNs exhibiting such non-ancestral missingness, see [30]. Note from this paper in particular that when interior variables are *systematically* missing, then there may well exist completely different explanations of the modelled system equally well-supported by the data—i.e., having the same observed likelihood—no matter how large the sample size. In such cases, it becomes imperative that strong prior information is introduced into the model to discriminate between these explanations. In particular, retaining approximate global independence without introducing such strong prior information is hopeless. Even in less extreme cases, there will usually be a necessary trade-off between retaining the clarity, interpretability, and in-house calculability of the resulting inference and its completeness. This judgement over the prior information used can only be expected to be possibly made by a well-equipped statistician or machine learner—usually only one of the academic team members.

Formal fast inferential Bayesian methodologies have already been developed and can help address the missingness of various types. This technology has already been successfully applied to a number of recent applications of less sensitive domains such as food security [31] and to energy systems [32]. The processes here are informed through composites of streaming state spacetime series whose components only inform the states describing various subsets of these states but not others. In this context, it is wise to try to ensure the inference is as formal as it can be without compromising its interpretability to the police. We would therefore recommend that one pragmatic solution is to follow the steps below:

1. Both teams accommodate all available open-source data whose likelihood separates into their shared CPTs in the way we describe above.
2. The academic team embed any further open-source data that destroy global independence but strongly inform the inference about some of the probabilities that otherwise would be extremely uncertain to both teams, and then update this joint distribution numerically [31] or with appropriate partial inference [32]. Then, the academic team approximate this posterior with a conjugate distribution—a product Dirichlet (or a mixture) exhibiting global independence across the CPTs with exponential (or more general) waiting times [29]. The academic team need to ensure that such an approximation is a close one in an appropriate sense or abort the accommodation of such data. These distributions are then translated in the library $L_{B}$.
3. The in-house team now refine their in-house distributions of their library of CPTs using the secure data available only to them, whose likelihood separates into their shared CPTs, replacing the independent posterior densities provided in $L_{A}$ to provide new distributions for these conditional probabilities in $L_{B}$.
4. All other unused in-house and open-source data are then simply used to inform and adjust the setting of priors only for future incidents as police deem appropriate.

### 5.3. Learning CPTs from Past Incidents and Across Different Graphs

To construct a library, it is important that, as far as possible, the distribution of the probabilities within the in-house library is supported by a formal Bayesian analysis. Here, the type of use to which the library will be put is critical when designing the level of complexity of the supporting formal estimation of its CPTs. Sometimes, libraries are built for the primary purpose of defining defences for a multitude of future incidents. One example of such a library of BNs is the support of networks of infrastructures to the increasing threats of flooding [21]. For such purposes where we need to predict a population of incidents, the dependence structure between estimated CPTs across different instances within the same class has a big impact on the assessment of the efficacy of different policy interventions. For example, the consequences when these future unfoldings across many different incidents are all identical will be very different to the case when each future instance is an independent realisation from the same conditional distribution.

However, when the primary use of the library is for real-time decision support, and thus to guide interventions that apply only to the next incident, inference about this dependence is not relevant. For this use—the one we focus on in this paper—the in-house team need to estimate only the expectations of the probabilities in the CPTs. This can often be conducted by quite naïve estimation and so is relatively easy for the academic team to guide remotely.

More explicitly, suppose the in-house team need to defend the next unfolding incident. Then inference simply corresponds to the implementation of standard propagation algorithms for a BN—here the relevant 2TDBN. We describe this more explicitly below. Note that the population of each of the CPTs in the library can be performed offline for each category of incident and suspect the police might encounter, and this is then plugged into the model of the current incident. The precise nature of this estimation—respecting its types and patterns of missingness—can be applied to each CPT, in turn using various techniques discussed elsewhere in this paper.

### 5.4. Learning Within an Ongoing Incident with a Known Suspected Perpetrator

Thus, suppose the police are faced with an unfolding incident and a suspect is then monitored in real time. We would recommend they follow the procedure outlined below:

1. Police match as closely as they can the category of incident they face to one they have catalogued within their library. Differentials between models in the library will often include the likely affiliation of the suspect, their broad intent, and their likely expression of attack—here the type of plot they are likely to execute (for example, a bomb attack or a vehicle attack). We assume that their library is rich enough to contain a plot model whose structure sufficiently matches the suspected incident.
2. Police then need to construct an appropriate set of CPTs that match the current incident. Depending on the maturity of the library, and depending on the category of incident and suspect, there will be CPTs of this 2TDBN populated with benchmark probabilities—whose expectations have been estimated from an archive of previous incidents and domain knowledge. Police embed these into the model whenever these benchmark generic expectations are available. Here, we have assumed this is the case for most of the CPTs. Note that after an incident has occurred, and particularly if this goes to court, then values of variables associated with tasks and phases will often become subsequently available. These can then be used to calibrate these otherwise latent variables, using the classifications mentioned above. This is especially useful for the matching of future incidents of the same or similar category to the current culminated plot, allowing a more accurate and calibrated translation of CPT entries from the developed library to ongoing cases.
3. Police then elicit the probabilities they need in-house to complete the probability model of the suspected perpetrator and incident based on private and covert information they have about the given suspect that goes beyond the category found in the library best matching the current case.
4. Such secure information is then embedded by the in-house team to sensitively adjust any entries in the CPTs to the current case, using the techniques they have developed through teaching from the academic team and through training in elicitation methods.
5. Police then simply apply this model to the current unfolding incident—embedding the specific routinely collected intensity data as well as sporadic intelligence data to update predictions concerning the efficacy of any interventions they contemplate making. How this works has been documented elsewhere [7,12].

We note that the scope of the application of the 2TDBN software to support decisions in this way depends on the existence of benchmark CPTs, estimated as described above. Of course, standard Bayesian diagnostics would be used in-house by police to determine whether or not there was information within the unfolding incident to suggest that the choice of model was inappropriate. If this is so, then the police will need to disregard the outputs of the support tool and reconsult the academic team.

Note that, within the Bayesian paradigm, if it is initially uncertain into which category a suspect falls, police can simply assign a probability across these different categories; for example, over different types of attack or over different intents and capabilities of the perpetrator—and use the unfolding intensity data to discriminate between these using standard Bayesian techniques for mixture modelling, as directed by the academic team. The only practical difficulty in setting this up is that they would then need to follow the steps outlined above for each of the plausible candidate models.

Of course, once the incident has concluded, further data relevant to the incident that was not previously accessible to the police will often become available in the form of court hearings and incident reports. Further resources may be employed to secure evidence in the prosecution proceedings, providing more data to the police than were available at the point of utilising the model for decision support. Police can use such data to update their probability distributions on the CPTs. Interestingly, if the suspect eventually turns out to be innocent, then data collected by police tagged to this person cannot be retained. However, they can first use this information to better inform the associated CPTs in-house. Notice that this would help mitigate the biases that might occur within open-source data because of the missingness of records about how innocent people might behave. Adjusting the utilised model in light of these new data sources ensures that, when the model returns to the library for this category of suspect and environment, it is as accurate and reliable as feasibly possible so that, if it is called upon as the best match for a future case, it is best equipped to mitigate the challenges of the missingness of current data for that future case.

### 5.5. Learning Through Intensities

Experience has taught us that much of the information that is to be kept secure concerns what police can covertly monitor, as well as the reliability of these data, to filter out the tasks the suspect might be engaged in. This impacts the application of the protocol above in two ways:

1. The guesses the academic team make about these aspects of the model will often be unreliable and so the in-house team will need to override a significant proportion of these variables, particularly by updating numerous entries of the corresponding CPTs.
2. The available streaming information concerning one incident and suspect can be very different to that available for another incident and suspect. Therefore, when populating the BN for a particular incident, this information will often need to be bespoke by default and hence entered by the police in-house.

To address this issue, we recommend that the academic team simply guess the *types* of tell-tale signs about such variables and provide a variety of probability settings for the corresponding CPT entries for the in-house team—reflecting the quality of the information from the hypothesised intensity measures—to match their scenario. These generic suggestions are particularly useful in helping the in-house team become aware of the implications of inferences they might make associated with past cases extended into further hypothetical cases in different settings via particular learning algorithms.

A key component of the plot model is that many model parameters, especially those specific to the suspect 𝒮, concern the speed at which the suspect can complete tasks and navigate phases of the plot. Experience has suggested that the misspecification of these parameters is not too critical provided these values are in the right ballpark. Parameters for which misspecification is the least critical are often the furthest in topological distance from the phase nodes. It can therefore be proved that these probabilities have less effect than those in other CPTs in the BN. See [33] for a detailed discussion about this issue.

It might superficially appear that such a process would be difficult to implement in real time. We end this section by using a hypothetical instance of a particular vehicle attack of the type described above to illustrate why this is proving not to be so.

### 5.6. Building on Generic Structures of Plots into BNs

The Bayesian model for the vehicle attack example was developed some time ago. The basic generic structural model we originally developed has survived scrutiny and has provided a useful initial template for many plots more generally. The phases of such attacks were conveyed directly to us across the firewall and, after confirming these through the academic literature on the subject, we were able to hardwire the corresponding non-zero states within the transition matrix for $w_{t}|w_{t-1}$. It was surprisingly easy for the academic team to discover the strong impact of the category of suspect (i.e., their background, including any sponsoring organisations, and their environment) on the speed of the development of a plot. The category of suspect was therefore clearly critical to the academic team in determining transition probabilities for an ongoing incident, modelled surprisingly well with open-source data. The academic team then communicated their information through their academic code. The main contribution of the in-house team was in providing summaries of their probabilistic beliefs that a particular category of suspect would abort an attack, and that one of their triaged population of suspects might be involved in a vehicle attack. The in-house team were then encouraged to input values (which in any case they would need to review on a case-by-case basis) specified directly by them.

Once we had elicited the task set as described above, we then needed to specify the DBN of the tasks conditional on $w_{0}.$ In our initial coded 2TDBN, we made the heroic assumption that component tasks evolved independently of each other. Although this assumption was extremely dubious, it nevertheless provided a learning algorithm that worked very well for predicting the phases of an attack. We would add that, in our current applications, we are embedding open-source information to estimate this sub-2TDBN directly. This suffers no missingness associated with security issues because such estimates can be based on benign observational data and extant sample surveys, although some components do admittedly suffer from missingness issues of a more conventional kind.

When a suspect is engaged in a phase of attack, the in-house team will typically have good information—based on past experience and internal intelligence about the particular suspect—about how they might act when in a particular phase to move to a subsequent phase. However, even in this case, the academic team can provide probabilities based on plausible logical guesses and open-source past case studies to provide the in-house team with a reasonable benchmark from which to vary their own specification.

If police need to make predictions about what might happen were they to intervene, they will need to revisit both the phase transition entries and the task probabilities associated with the active states after any such contemplated intervention. For example, they may choose to arrest the suspect when the probability that the suspect has reached a sufficiently advanced phase of the attack is high enough to secure such an arrest.

Training for the in-house team to embed the additional CPTs needed for such an embellishment is actually straightforward to provide, given that the academic team ensure that the 2TDBN is *adversarially causal*. This is a delicate matter for the academic team beyond the scope of this paper. For a detailed recent discussion about how the academic team might do this in this context, see [13]. However, once the underlying graphical framework has been confirmed as causal for the academic model, then this property will translate seamlessly to the in-house replicate. So, with regard to missingness and firewall estimation, this embellishment introduces no new challenges once the graphical framework is in place. The intensity measures associated with each of these tasks can be guessed by the academic team, as discussed above.

## 6. Discussion

Having now developed this protocol, our academic team are currently populating a number of different libraries of the type we have described above with various in-house teams. We will report additional findings later on in this developmental process. However, even at this early stage, we are able to report both some challenges and some advancements encountered when applying this graphically enhanced technological transfer.

We first discuss some of these challenges and limitations of the methodology as presented in this paper. First, it is important to note that, while the BN is a powerful structural model, it is not the only class of model we can use. For many classes of criminal activity, it is not the most efficient. Even for plots described here and in [7], we can argue for not using a 2TDBN as a template, as used in this paper, but rather a particular customised hybrid graph with different semantics. We will provide a detailed description of such customised hybrid graphs as well as a demonstration of how they can be applied to efficiently model plots in a subsequent paper. These graphs are able to depict directly, and thus more elegantly and transparently, much more of the acknowledged structural information described in Section 3. For example, through such constructions, we can directly represent the task sets as well as impossible phase transitions. The protocols above are expressed for the more generic family of 2TDBNs but translate directly to this more customised family of models. This translation is especially important for the study of applications for which the structural dynamics are poorly rather than inefficiently expressed by a DBN. Such applications include bot or incursion attacks [13], as opposed to the plot models we see here. What we would lose, however, in utilising this customised family of models, is the valuable resource of already extant suites of accessible quality-controlled software that supports BN-based inference. Therefore, we recommend the choice of an appropriate structural framework to be made on a case-by-case basis. As police are in any case constructing their own bespoke code behind the firewall, it is often helpful to base libraries around these more customised graphs, though we do not focus on these within the development of libraries of models in this paper.

We are furthermore limited thus far by the elicitation techniques we have used in practice in constructing these libraries of models. The choice of elicitation techniques depends on the skills and resources available to both the academic and in-house teams. In our experience of developing these models, only a narrow scope of elicitation techniques have been utilised, as there has been a lack of diversity in the elicitation backgrounds held by academics who have so far been involved in this type of co-creation. We aim to apply and analyse a more diverse range of elicitation methods, as discussed in Section 4.2.1, in the future. Finally, we report that this methodology does require a substantial commitment from both the academic and in-house teams. In such co-creation, it is vital that both teams provide sufficient input, skills, and resources in a timely manner for progress to be made at a good rate. This is especially true in the early days of a co-creation project, and it can be hard to reach a point in the process where the in-house team begin to reap worthwhile rewards from ownership of large libraries of accurately and finely constructed models.

It is clear in our experience that there are significant benefits from having developed large libraries of models. For example, as the libraries of 2TDBNs—or other bespoke graphs—are built up, much more sophisticated methodologies can be developed to estimate the necessary CPTs, in turn reducing the degree of reliance on elicitation methods for defining the quantitative structure of the model. Reduced reliance on elicitation methods is a clear benefit, as such methods can easily lead to catastrophic inaccuracies being built into a model if the elicitation is not very carefully structured and carried out [10]. Further, as the library grows in size, it becomes more likely that a new, unfolding plot will be sufficiently similar to a culminated plot for which a model in the library has been specifically made. Therefore, this extant model is able to be used both to aid the quick construction of a new bespoke model for the ongoing plot (which may just need a handful of amendments from the extant model) and to aid decision-making by police.

The ability to utilise an extant model in developing a new, bespoke model for an unfolding plot is a significant benefit to the police due to the potentially rapid developments of particular plots. Not only can a single extant model be used to aid the development of a new model for an unfolding plot, but we can also extract features from several models to guide the creation of the new model. The modularity of a BN allows us to extract particular structural and quantitative features from several networks which can then be adjusted and pasted together to form the basis for the new model. When a library of extant models becomes large, we are able to find components of extant models that suit the equivalent components of the new model being built for a new plot. In particular, the ability to find matching CPTs for a new model being developed is of significant benefit to the police teams as this element of the model creation is often the most crucial in ensuring model accuracy, and as many structural components will naturally remain the same across many plots. This modularity property being used to efficiently and locally update BNs can be seen in [2].

The research presented in this paper naturally sits at an exciting intersection of several fast-moving research areas. For example, recent vigorous developments in adversarial risk analysis [34,35], where causal hypotheses are plausible—as they are for plot models—and are being harvested for use within this domain of crime prevention [13]. However, we have only just scratched the surface of the potential of this symbiosis, and much fruitful work remains to be conducted to exploit this extant technology and theory—in particular, to support, through the types of co-creation described above, the in-house libraries of crime incidents needed by the police.

One point we particularly hope to have illustrated above is how—by employing a subjective Bayesian approach—sparse, contaminated, and sporadic data can nevertheless be combined with informed expert judgements and previously constructed models to build robust in-house dynamic probability models for unfolding plots. These can then be used by police in their pursuit of criminals who they suspect are intent on extreme violence against the general public. It is clear that terrorism intervention is not the only domain of application for which the protocols we describe in this paper are of significant benefit; these protocols could also be applied to asset, health, and national infrastructure models, among many others. Within such contexts, missingness stemming from the high security of personal and national data is an inherent feature of such analyses, but this is an issue we are able to overcome by developing libraries of bespoke models following the co-creation protocols presented in this paper.

## Data Availability

Not applicable.

## Abbreviations

The following abbreviations are used in this manuscript:

BN	Bayesian network
CPT	Conditional probability table
DAG	Directed acyclic graph
DBN	Dynamic Bayesian network
2TDBN	Two time-slice dynamic Bayesian network
MO	Modus operandi
SCM	Structural causal model
SEU	Subjective expected utility

## Appendix A. Structure of the Phase Transition Matrix

The explicit form of the transition matrix between phases and its parameteri- sation—which is retained even after intervention—is given below. For the first phase, which is absorbing, we have that, for all t≥1

$$p_{t}^{w}\left(w_{jt}|w_{0t-1}\right)=\left\{\begin{array}{cc} 1 & j=0\\ 0 & j\neq 0 \end{array}\right.$$

while for i=1,2,…,m and t≥1

$$p_{t}^{w}\left(w_{jt}|w_{it-1}\right)=\left\{\begin{array}{cc} q_{ti}^{\prime } & j=0\\ \left(1-q_{ti}^{\prime }\right)q_{ti} & 1\leq i=j\leq \;m\\ \left(1-q_{ti}\right)\left(1-q_{ti}^{\prime }\right)p_{tij}^{w} & 1\leq i\neq j\leq m,j\in E_{i}\\ 0 & 1\leq i\neq j\leq m,j\notin E_{i} \end{array}\right.$$

Here, $q_{ti}^{\prime }$ denotes the probability that the agent will abort the plot, and $q_{ti}$ the probability that the agent will remain in the same phase, both when in state *i* and transitioning from time t−1 to time *t*. The probability $p_{tij}^{w}$ denotes the probability the next phase will be $w_{j}$ when in phase $w_{i}$ at time t−1, given the agent leaves their current phase and does not abort the plot. $E_{i}$ are the states that can be reached from phase $w_{i}$ in one time step. Note that when $E_{i}=\left\{w_{j^{*}}\right\}$, so that a criminal can only transition from phase *i* to a single phase $w_{j^{*}}$, then by definition $p_{tij^{*}}^{w}=1$ and thus does not need to be elicited.
